# Characterizing Interhospital Variability in Neurosurgical Interventions for Patients with Mild Traumatic Brain Injury and Intracranial Hemorrhage

**DOI:** 10.1089/neur.2022.0078

**Published:** 2023-03-17

**Authors:** Alessandro Orlando, Josef Coresh, Matthew M. Carrick, Glenda Quan, Gina M. Berg, Laxmi Dhakal, David Hamilton, Robert Madayag, Carlos H. Palacio Lascano, David Bar-Or

**Affiliations:** ^1^ Department of Epidemiology, Bloomberg School of Public Health, Johns Hopkins University, Baltimore, Maryland, USA.; ^2^ Department of Trauma Services, Medical City Plano, Plano, Texas, USA.; ^3^ Department of Trauma Services, Swedish Medical Center, Englewood, Colorado, USA.; ^4^ Department of Trauma Services, Wesley Medical Center, Wichita, Kansas, USA.; ^5^ Department of Trauma Services, Penrose Hospital, Colorado Springs, Colorado, USA.; ^6^ Department of Trauma Services, St. Anthony Hospital, Lakewood, Colorado, USA.; ^7^ Department of Trauma Services, South Texas Health System McAllen, McAllen, Texas, USA.; ^8^ Injury Outcomes Network, Englewood, Colorado, USA.

**Keywords:** epidemiology, intracranial hemorrhage, mild, neurosurgical intervention, National Trauma Data Bank, traumatic brain injury, variability

## Abstract

The objective of this study was to quantify nation-wide interhospital variation in neurosurgical intervention risk by intracranial hemorrhage (ICH) type in the setting of mild traumatic brain injury (mTBI). This was a retrospective cohort study of adult (≥18 years) trauma patients included in the National Trauma Data Bank from 2007 to 2019 with an emergency department Glasgow Coma Scale score 13–15, diagnosed ICH, no skull fracture. The primary outcome was neurosurgical intervention. Interhospital variation was assessed by examining the best linear unbiased predictors (BLUPs) obtained from mixed-effects logistic regression with random slopes and intercepts for hospitals and covariates for time and 14 demographic, injury, and hospital characteristics; one model per ICH type. Intercept BLUPs are estimates of how different each hospital is from the average hospital (after covariate adjustment). The study population included 49,220 (7%) neurosurgical interventions among 666,842 patients in 1060 hospitals. In 2019, after adjusting for patient case-mix and hospital characteristics, the percentage of hospitals with hemorrhage-specific neurosurgical intervention risk significantly different from the average hospital was as follows: isolated unspecified hemorrhage (0% of 995 hospitals); isolated contusion/laceration (0.54% of 929); isolated epidural hemorrhage (0.39% of 778); isolated subarachnoid hemorrhage (0.10% of 1002); multiple hemorrhages (2.49% of 963); and isolated subdural hemorrhage (16.25% of 1028). In the setting of mTBI, isolated subdural hemorrhages were the only ICH type to have considerable interhospital variability. Causes for this significant variation should be elucidated and might include changing hemorrhage characteristics and practice patterns over time.

## Introduction

There are an estimated 240,000 mild (mTBI) traumatic brain injuries (TBIs) with intracranial hemorrhage (ICH; complicated mTBI) observed each year in the United States.^1–4^ These injuries are considered “mild” according to their neurological status, but are considered serious with regard to their potential need for an emergent neurosurgical intervention. As such, multiple management guidelines suggest that these patients receive a neurosurgical consultation,^5–7^ often resulting in an interhospital transfer.^8,9^ These guidelines provide general management recommendations and supporting literature; nevertheless, each acknowledges the limited evidence and contains non-specific language.

The limitations of current mTBI guidelines translate into neurosurgeons making individual decisions on who to intervene surgically and sometimes results in the disregarding of guideline recommendations^10^; these factors—and others—engenders practice variations. From 2014 to 2015, van Essen and colleagues surveyed 68 hospitals across Europe about their neurosurgical decision-making practices.^11^ They discovered wide variability in neurosurgical practice regarding the decision for neurosurgical intervention, including varied methods for estimating the size of the lesion with head imaging (e.g., visually, two-dimensional measurements, or volume estimation), varied lesion-size thresholds for neurosurgical intervention (e.g., 15 mm, 5 mm, lesion thickness > skull thickness), and multiple thresholds for intracranial pressure prompting decompressive craniotomy (ranging from 30 mm Hg to “not standardized”). In a 2022 systematic review by Barrett and colleagues, the gamut of neurosurgical decision making in patients with TBI was also highlighted.^12^ Further, there have been publications highlighting practice variation in the setting of severe TBI and across severities of TBI, each showing the wide multitude of factors—other than a patient's injury—that are incorporated into the decision to proceed with a neurosurgical intervention.^10,13^

A limitation of the current literature highlighting variations in neurosurgical practice is that it does not stratify the variation by the type of ICH. It has been shown that the proportion of patients with complicated TBI receiving neurosurgical intervention varies by the type of ICH^14,15^; thus, there are varied neurosurgical considerations depending on the type of ICH. If neurosurgical practice variations are averaged across all hemorrhage types, it could mask high or low practice variations in specific hemorrhage types. Other limitations of the current literature on neurosurgical practice variations include the paucity of studies, often with simple cross-sectional assessments of practice patterns, or that the quantification of the variability is not of primary interest.^13,16^

Recently, a multi-center observational study across Europe had the objective to quantify the variability in neurosurgical decision making in patients with TBI.^17^ This study was limited to patients presenting with subdural hemorrhages (SDHs) and included patients across the severity spectrum of TBI, making it difficult to disentangle the relationship between severity of TBI and variation in neurosurgical interventions. The objective of the current study was to quantify interhospital variability in neurosurgical interventions in the United States, focusing on patients with mTBI and stratifying by the type of ICH.

## Methods

This was a retrospective cohort study of patients admitted to U.S. hospitals with complicated TBI. We used the American College of Surgeons (ACS) National Trauma Data Bank (NTDB), the largest database of trauma patients in the United States. Patients were included in the study if they: 1) were admitted to from 2007 to 2019; 2) were 18–89 years of age; 3) had an emergency department Glasgow Coma Scale (GCS) score of 13–15; and 4) presented with at least one of the following trauma-related initial encounter International Classification of Diseases [ICD], Ninth Revision (ICD-9) or Tenth Revision (ICD-10) diagnosis codes for an ICH: contusion/laceration of parenchyma or cerebellum, SDH, epidural hemorrhage (EDH), subarachnoid hemorrhage (SAH), or unspecified ICH. Patients were excluded for 1) having a skull fracture and 2) any missing covariate data. These data were publicly available and did not contain identifiable information; this study was not considered human subjects research.^18,19^

### Covariates and outcome

We used the following patient- and hospital-level covariates from the NTDB: year of hospital admission; age (years); biological sex (male, female); self-identified race (Asian, Black, White, or Other); payment method (Medicaid, Medicare, private/commercial, or self-pay); an anonymized hospital identifier; hospital bed size (≤200, 201–400, 401–600, or >600); ACS trauma designation level (1, 2, 3, 4, or undesignated); number and type of ICH (isolated contusion/laceration of parenchyma or cerebellum, isolated SDH, isolated EDH, isolated SAH, isolated unspecified ICH, and multiple types of ICHs); interhospital transfer (yes/no); mechanism of injury (fall, motorcycle accident, motor vehicle accident, struck by/against, transport [other], and other); emergency department GCS score; Injury Severity Scale (ISS) score; injury type (blunt, penetrating, burn, or other/unspecified); intent of injury (assault, self-inflicted, unintentional, undetermined, or other/unspecified); presence of pre-injury anticoagulant, antiplatelet, thrombin inhibitor, or thrombolytic agent (chronic aspirin use excluded); and admission systolic blood pressure (mm Hg), pulse (beats/min), and respiratory rate (breaths/min).

Documented neurosurgical intervention was the primary outcome as determined by ICD-9/-10 procedure codes. The study spanned time frames when ICD-9 and ICD-10 codes were used. As such, we harmonized ICD-9 and -10 procedure codes. Because ICD-10 codes are more specific than ICD-9 codes, we converted all ICD-10 procedure codes to ICD-9 procedure codes; ICD-10 to ICD-9 mappings were provided by the Center for Medicare and Medicaid Services.^20^ Neurosurgical procedures were defined using ICD-9 codes 1.00–2.99. See the Supplementary Material for a full mapping of all neurosurgical intervention procedure codes used in this study.

### Statistical analyses

The main analysis quantifying interhospital variability in the use of neurosurgical interventions used a mixed-effects logistic regression model with random intercepts and slopes with an unstructured covariance model. This model was adjusted for all covariates previously outlined; separate models were run for each ICH type.

A best linear unbiased predictor (BLUP) was calculated by the model for each hospital and is a weighted estimate of how much a hospital deviates from the average hospital, after adjusting for the fixed effects (covariates); weighting (a.k.a., shrinkage estimate) is based on the amount of variance in the data contributed by a hospital. If a hospital's data had low variance, then the model allowed that hospital to deviate more from the average hospital, if indeed it was different. However, if a hospital's data were highly variable, then the model decreased the amount that hospital could deviate from the average hospital. In this study, a positive BLUP indicated that a hospital had an estimated adjusted odds of neurosurgical intervention higher than the average hospital, whereas a negative BLUP indicated that a hospital had an estimated adjusted odds of neurosurgical intervention lower than the average hospital. The standard error of each hospital's BLUP was used to calculate 95% confidence intervals and assess whether each hospital was significantly different from the average hospital: BLUP ±1.96*(standard error of BLUP), with a null hypothesis that a BLUP was equal to 0.

BLUPs for the model intercepts and slopes were used to quantify the amount of interhospital variability in neurosurgical intervention. The intercept BLUP quantified how different a hospital's use of neurosurgical intervention was from the average hospital. To characterize interhospital variability at different years, we ran each model three times, each time using a different centering of the year variable: centering in 2007, 2014, and 2019. This characterized interhospital variability at the beginning, middle, and end of the study period. As an additional metric of interhospital variation, we calculated the median odds ratio stratified by ICH type using the 2019-centered model, which quantified the median fold difference in odds of neurosurgical intervention comparing the same patient in two randomly chosen hospitals in 2019.^21^

Pearson's correlation analyses were conducted to determine the between ICH correlation of the intercept BLUPs from the model using the 2019-centered time variable. This analysis determined the extent to which a hospital's ICH-specific neurosurgical intervention use was correlated with the neurosurgical intervention use in other ICH types (e.g., if a hospital operated above average for one hemorrhage type, did they also operate above average from another hemorrhage type?).

To characterize the interhospital variability in how hospitals changed over time vis-à-vis their use of neurosurgical interventions, we used the slope BLUP from a model using the 2014 time-centered variable, because this provided more stable estimates of each hospital's slope. Because the average hospital had decreasing odds of neurosurgical intervention over time, a negative slope BLUP indicated that a hospital's odds of neurosurgical intervention decreased faster than the average hospital; positive slopes indicated a slower than average decreasing odds of neurosurgical intervention. All hypothesis tests were two-tailed with an alpha of 0.05, and SAS software was used (version 9.4; SAS Institute Inc., Cary, NC).

## Results

The final study population consisted of 666,842 patients and 1060 hospitals. A detailed description of the study population can be found elsewhere.^22^ Briefly, 36% (238,419) of patients presented with an isolated SDH, 24% (159,220) with multiple ICH types, 24% (157,414) with isolated SAHs, 9% (60,315) isolated unspecified hemorrhages, 7% (43,695) with isolated lacerations/contusions, and 1% (7,779) with isolated EDHs; this was consistent with a detailed exploration of time trends in this population.^22^ Average age (standard deviation; SD) was 63.3 (19.44) years, 58% were male, and 81% were White. Unintentional falls were the leading mechanism of injury (63%), with 78% having an emergency department GCS of 15 and an average ISS score of 14.65 (7.74). Nearly 40% of patients were interhospital transfers.

At least one neurosurgical intervention was documented in 49,220 patients. The observed median (interquartile range) hospital-specific neurosurgical intervention percentage was 0.0% (0.0–14.8%) for isolated EDHs, 0.0% (0.0–1.8%) for isolated lacerations/contusions, 5.6% (0.0–8.3%) for multiple hemorrhage types, 0.0% (0.0–0.8%) for isolated SAHs, 10.4% (0.0–15.4%) for isolated SDHs, and 0.0% (0.0–2.4%) for isolated unspecified hemorrhages.

Marginal estimates from the multivariate mixed-effects logistic regression model suggested that all ICH types showed significant decreases in adjusted odds of neurosurgical intervention across the 13-year period, except for isolated EDHs and lacerations/contusions; these two ICH types were not shown to have significant changes over time in their odds of neurosurgical intervention.^22^

**FIG. 1.**
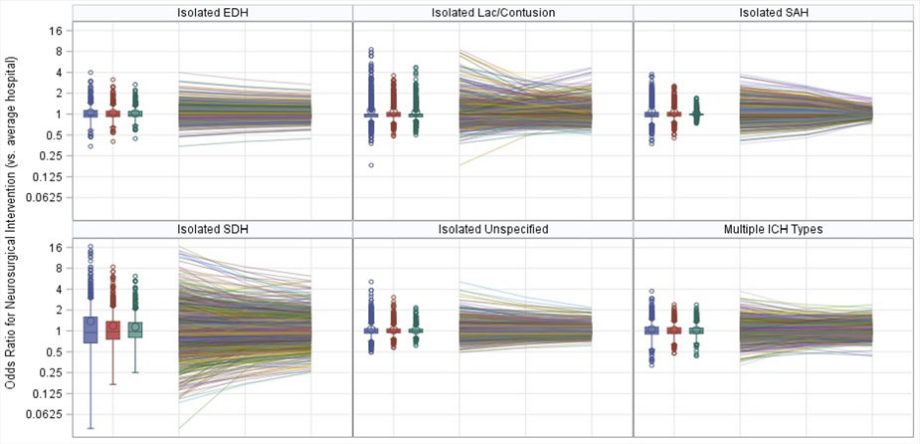
Long-term change in interhospital variation in neurosurgical intervention, stratified by hemorrhage type. Box-and-whisker plots and spaghetti plots showing longitudinal change in interhospital variation in neurosurgical intervention, stratified by ICH type. Each observation is one hospital. Hospital-specific best linear unbiased predictors were transformed into odds ratios for neurosurgical intervention, comparing each hospital to the average hospital. An odds ratios above/below 1 indicates that a hospital had a higher/lower than average adjusted odds of neurosurgical intervention. All hemorrhage types showed decreasing interhospital variation over time. Isolated SDHs had the largest amount of interhospital variation in neurosurgical interventions. EDH, epidural hemorrhage; ICH, intracranial hemorrhage; Lac, lacerations; SAH, subarachnoid hemorrhage; SDH, subdural hemorrhage.

Random intercepts from the mixed-effects models indicated that nearly all hospital-specific adjusted odds ratios for neurosurgical intervention (compared to the average hospital) showed decreasing variability over time (Fig. 1). Additionally, SDs of the intercept BLUPs were dependent on ICH type (Table 1). Isolated SAHs showed the largest relative decrease over time in the SD of hospital-specific intercept BLUPs for neurosurgical intervention. By 2019, hemorrhage-specific SDs were grouped into three categories of variability: high (isolated SDHs); medium (isolated laceration/contusions); and low (isolated epidural, subarachnoid, unspecified, and multiple hemorrhage types).

**Table 1.** Standard Deviations of Hospital-Specific Best Linear Unbiased Predictor of Neurosurgical Intervention

Time-centered model		2007	2014	2019	
BLUP SD	Total Hospitals^a^	Intercept	Intercept	Intercept	Slope
Isolated Epidural	778	0.25	0.21	0.19	0.01
Isolated Laceration/Contusion	929	0.36	0.25	0.31	0.03
Multiple Hemorrhage Types	963	0.28	0.20	0.19	0.01
Isolated Subarachnoid	1002	0.27	0.21	0.17	0.04
Isolated Subdural	1028	0.76	0.54	0.45	0.01
Isolated Unspecified	995	0.24	0.18	0.15	0.02

^a^ Not all hospitals contributed patient data to each intracranial hemorrhage type.

BLUP, best linear unbiased predictor; SD, standard deviation.

Table 2 shows the number and percentage of hospitals with significantly different adjusted odds of neurosurgical intervention, compared to the average hospital. In 2019, isolated SDHs had the largest proportion of hospitals with significantly different odds of neurosurgical intervention (16.25%), compared to the average hospital, and isolated unspecified hemorrhages had the lowest (0%). Further, in 2019, the median odds ratio was 1.59 for isolated EDHs, 2.35 for isolated lacerations/contusions, 1.57 for isolated SAHs, 1.77 for isolated SDHs, 1.57 for isolated unspecified hemorrhages, and 1.41 for multiple hemorrhages.

**Table 2.** Number of Hospitals with Odds of Neurosurgical Intervention Significantly Different from the Average Hospital

N (%)	Total hospitals^a^	2007	2014	2019
Isolated Epidural	778	4 (0.51%)	6 (0.77%)	3 (0.39%)
Isolated Laceration/Contusion	929	14 (1.51%)	12 (1.29%)	5 (0.54%)
Multiple Hemorrhage Types	963	40 (4.15%)	63 (6.54%)	24 (2.49%)
Isolated Subarachnoid	1002	14 (1.40%)	17 (1.70%)	1 (0.10%)
Isolated Subdural	1028	199 (19.36%)	277 (26.95%)	167 (16.25%)
Isolated Unspecified	995	9 (0.90%)	9 (0.90%)	0

^a^ Not all hospitals contributed patient data to each intracranial hemorrhage type.

**FIG. 2.**
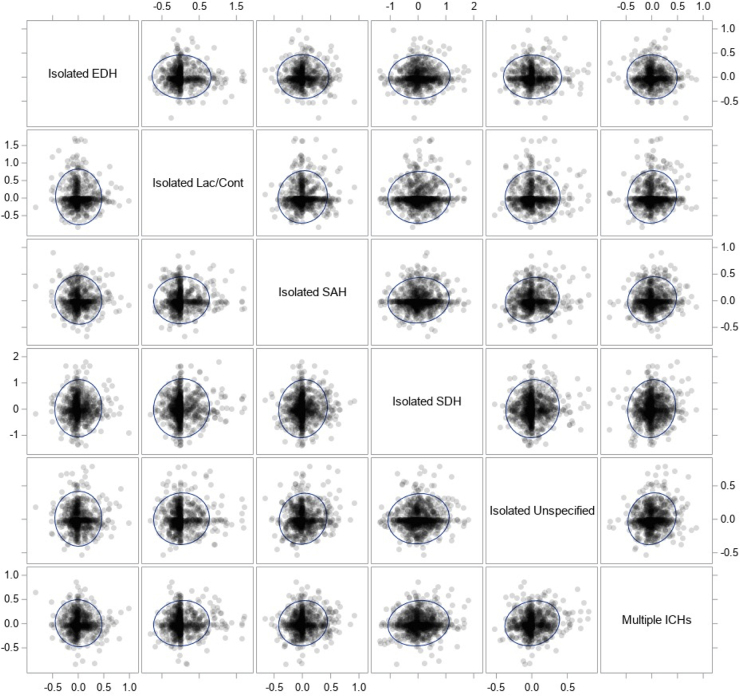
Scatterplot matrix of each hospital's hemorrhage-specific 2019 intercept BLUP. Scatterplots of intercept BLUPs from the model using the 2019-centered time variable do not suggest a strong correlation between ICH types in how a hospital's neurosurgical intervention use compares to the average hospital. Each dot is one hospital. BLUP, best linear unbiased predictor; EDH, epidural hemorrhage; ICH, intracranial hemorrhage; Lac, lacerations; SAH, subarachnoid hemorrhage; SDH, subdural hemorrhage.

Figure 2 shows the correlation matrix of each hospital's hemorrhage-specific 2019 intercept BLUP, and Table 3 shows the Pearson correlation coefficients and respective *p* values for each comparison. Correlation analyses suggested significant positive correlations between all hemorrhage-specific intercept BLUPs, except isolated EDHs and isolated laceration/contusions.

**Table 3.** Correlation Matrix for Hemorrhage-Specific Best Linear Unbiased Predictors for Hemorrhage-Specific Intercepts, Using 2019-Centered Model

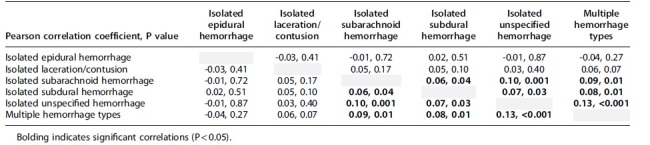

**FIG. 3.**
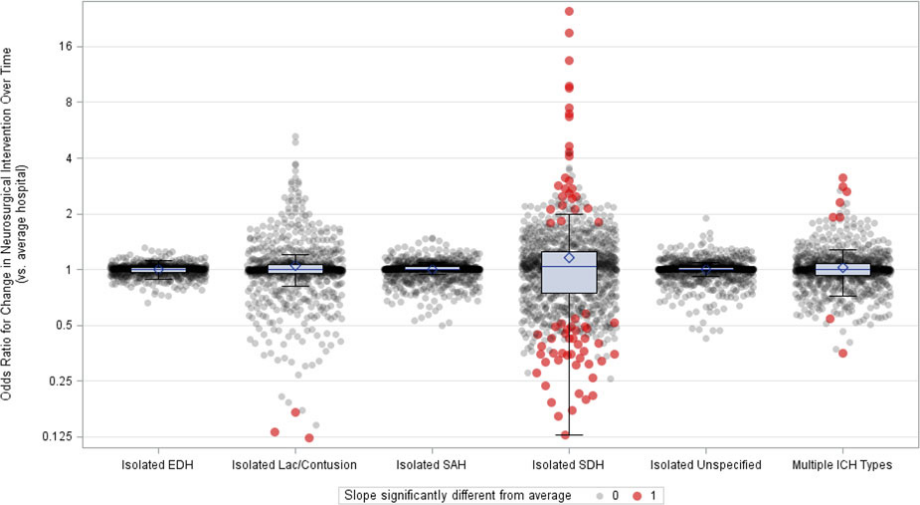
Interhospital variability in long-term change in neurosurgical interventions. Box-and-whisker plots showing interhospital variation in longitudinal change in neurosurgical intervention, stratified by ICH type. Data shown are hospital-specific best linear unbiased predictors for slope transformed into odds ratios (per 12 years) for neurosurgical intervention, comparing each hospital to the average hospital. An odds ratio above/below 1 indicates that a hospital had a slower/faster than average 12-year change in adjusted neurosurgical intervention. Isolated SDHs had the largest interhospital variation in how hospitals changed over time with respect to long-term relative trends in neurosurgical interventions. EDH, epidural hemorrhage; ICH, intracranial hemorrhage; Lac, lacerations; SAH, subarachnoid hemorrhage; SDH, subdural hemorrhage.

Random slopes obtained from the 2014-centered mixed-effects models suggested that SDs of the random slope BLUPs were smaller than SDs of the random intercept BLUPs (Table 1). Isolated EDHs had the smallest SD in random slopes, whereas isolated SDHs had the largest. Of all the hospitals contributing data, 6.7% of 1028 hospitals had significantly different changes in the adjusted odds of neurosurgical intervention for isolated SDHs over time, compared to the average hospital; 0.8% of 963 hospitals for multiple hemorrhage types; and 0.3% of 929 hospitals for isolated laceration/contusions (Table 2; Fig. 3). For isolated EDHs, isolated SAHs, and isolated unspecified hemorrhages, no hospital had a significantly different change in the odds of neurosurgical intervention over time, compared to the average hospital.

## Discussion

This study presents novel data characterizing interhospital variability in neurosurgical intervention and variability in how odds of neurosurgical intervention have changed over time in a representative population of patients presenting with complicated mTBI in the United States. These data suggest that isolated SDHs had the largest interhospital variability in odds of neurosurgical intervention, and that there was little interhospital variability in how the ICH-specific odds of neurosurgical interventions have changed over time. The results of this study are important for future research of complicated mTBIs and for understanding the current landscape of neurosurgical interventions in the United States.

When management guidelines for complicated mTBIs are often supplanted by clinical experience, it is generally accepted that there will be practice variation with respect to neurosurgical interventions. Yet, there is a serious paucity of literature properly quantifying the magnitude of neurosurgical practice variation in the United States, specifically in patients with complicated mTBI. van Essen and colleagues quantified the variability of neurosurgical intervention in patients with complicated mTBI in Europe and observed considerable practice variation.^11,16,17^ For example, when 60 neurosurgeons were presented with the head computed tomography and a case vignette of a 79-year-old male with a GCS of 14, a 20-mm left-sided acute SDH, and 9 mm of midline shift, 19 (32%) indicated that they would not perform an operation on this patient, but 41 (68%) said that they would operate.^16^ Additionally, in a CENTER-TBI (Collaborative European Neurotrauma Effectiveness Research in Traumatic Brain Injury) prospective cohort study on 1160 patients with acute SDH, the median odds ratio for neurosurgical intervention was 1.8, indicating a 1.8-fold median difference in odds of neurosurgical intervention between 2 identical patients in two randomly chosen hospitals.^17^ Nevertheless, these characterizations of variability of neurosurgical intervention were done in a European population and were limited to acute SDHs of varying severities.

Despite the geographical differences between the large European van Essen and colleagues study and the current U.S. study, both reported similar median odds ratios for SDH neurosurgical interventions: ∼1.8. The current study adds to the literature by adumbrating the landscape of variability of neurosurgical interventions in complicated mTBIs in the United States, uniquely characterizing the variability according to the type of ICH.

In 2019, isolated SDHs had the largest variability in the BLUPs for neurosurgical intervention. From another perspective, isolated SDHs had the second largest median odds ratio in 2019 (1.77), whereas isolated lacerations/contusions had the largest median odds ratio (2.35). These seemingly inconsistent findings can be reconciled by recalling two aspects of each hemorrhage type: the percentage of patients receiving a neurosurgical intervention and the interhospital variability about that percentage (i.e., SD of the intercept BLUPs). Isolated lacerations/contusions had the second *largest* SD of the intercept BLUP and also had the second *smallest* neurosurgical intervention percentage (1.3%); therefore, this hemorrhage type had small absolute odds of neurosurgical intervention, but moderately large variability, leading to large *relative* differences in median odds between hospitals. Thus, the median odds ratio, variability of the BLUPs, and absolute proportion receiving neurosurgical interventions should be considered jointly when making inferences about the variability of neurosurgical interventions.

Further complicating the objective of quantifying interhospital variability of neurosurgical intervention is the need to establish whether a hospital is significantly different from the average hospital given the variance of its data. We used the standard error of each hospital's intercept BLUP to establish significance, allowing for the calculation of how many hospitals were considered significantly different from average, after adjusting for important confounding variables. Our results suggested that isolated SDHs had the largest proportion of hospitals with significantly different odds of neurosurgical intervention (16.25%), compared to the average hospital; the remaining ICH types had minimal proportions of hospitals being significantly different from average (all <2.5%). Taken together with the other results, it is clear that the largest interhospital variability in neurosurgical interventions for patients with complicated mTBI occurs in the setting of isolated SDHs.

The reasons for this large variability are undoubtedly multi-factorial,^23^ but are unlikely to be attributable to the demographic, clinical, and hospital characteristics controlled for in our analyses. Differing hospital care guidelines and varying levels of neurosurgical coverage and expertise are more plausible explanations for the variability in neurosurgical intervention. On the other hand, these potential explanations would not be expected to be unique to SDHs, yet isolated SDHs were uniquely variable. Instead, perhaps the differences in hemorrhage-specific neurosurgical variability highlight the differences in how each ICH can present or evolve throughout hospitalization, and how those differences impact the decision for neurosurgical intervention. For example, SDHs might have higher varied initial presentations or evolutions throughout hospitalization, compared to isolated SAHs, leading to increased variation in neurosurgical decisions. Nevertheless, the exact reasons remain unknown.

Assessing the correlation of the hemorrhage-specific 2019-centered intercept BLUPs allowed us to answer the question: If a hospital's neurosurgical staff intervened more than average for one ICH type, did they intervene more than average for other hemorrhage types, after controlling for the 15 demographic, injury, and hospital characteristics? If neurosurgical groups who operated higher than average did so for all ICH types, then we would expect a high correlation between hemorrhage-specific intercept BLUPs. Although there were many statistically significant correlations between ICH types, all had Pearson correlation coefficients no larger than 0.13, indicating weak correlations. Thus, if a hospital operated higher than average for one ICH type, it tells us little about how that hospital's neurosurgical intervention rate ranked with respect to other ICH types; each ICH type is unique in how frequently each hospital neurosurgically operates.

These results have important implications for future research in patients with complicated mTBI. First, the growing focus on the creation of predictive models for neurosurgical intervention in this patient population underscores the need to better incorporate an understanding of interhospital variation.^14,24–28^ Our current study reaffirms the need to externally validate predictive models of neurosurgical intervention in multiple centers, given that the variability in neurosurgical decision making is non-ignorable and differs by the type of ICH. Nevertheless, it is important to take steps to explain and understand the variability between hospitals. Factors not examined in the current study, including hemorrhage characteristics (i.e., hemorrhage size and location), might partially explain the interhospital variability in neurosurgical interventions.^11^

The current study has multiple limitations that need to be considered when making conclusions on the data. First, the NTDB is a voluntary data set that is a convenience sample of trauma patients across the United States. It is the responsibility of each hospital to ensure that the data contributed are of high quality. Because we were limited to the variables available in the NTDB, we cannot dismiss the possibility of residual and unmeasured confounding in our analyses. Additionally, the NTDB redacts ages >89 years; therefore, our conclusions are based on persons 18–89 years of age. It is possible that there is less or more overall variability in neurosurgical interventions in those ≥90 years; however, without more detailed age data, it is unknown whether the current results over- or underestimate the variability.

The main analyses used a mixed-effects logistic regression model to estimate the neurosurgical intervention proportion for each hemorrhage type and also for each hospital. This method sacrifices variance, in exchange for less bias; hospitals with more variance in their neurosurgical intervention data are biased toward the population average proportion receiving neurosurgical intervention. It could be argued that this method underestimates the true interhospital variation in neurosurgical interventions. Though true, our methodology also avoids overestimating variability by not equally weighting hospitals with inconsistent or highly variable data. We chose to underestimate variability, rather than overestimate. Despite these limitations, the study remains impactful because of its strengths. The NTDB is the largest compilation of trauma patients in the United States and—in more recent years—is representative of ACS-verified trauma centers. Additionally, we used deidentified hospital identifiers that allowed us to cluster patients within hospitals and make use of each hospital's repeated data across time.

## Conclusion

This nationwide study presents results showing the large interhospital variability of neurosurgical interventions in patients with complicated mTBI in the United States, and how that variability differs based on the type of ICH. These results indicate that after adjustment for multiple demographic, injury, and hospital characteristics, isolated SDHs had the largest hemorrhage-specific interhospital variability in neurosurgical interventions, though all other hemorrhage types examined had non-ignorable levels of interhospital variability. Future research should identify characteristics that account for the large interhospital variation found in this current study and strategize methods for increasing comparability in neurosurgical decision making across hospitals.

## Supplementary Material

Supplemental data

## Abbreviations Used

ACS: American College of Surgeons
BLUPs: best linear unbiased predictors
EDH: epidural hemorrhage
GCS: Glasgow Coma Scale
ICD: International Classification of Diseases
ICD-9: International Classification of Diseases, Ninth Revision
ICD-10: International Classification of Diseases, Tenth Revision
ICH: intracranial hemorrhage
ISS: Injury Severity Scale
mTBI: mild TBI
NTDB: National Trauma Data Bank
SAH: subarachnoid hemorrhage
SD: standard deviation
SDH: subdural hemorrhage
TBI: traumatic brain injury
