# Supplementary material for: Characterizing Interhospital Variability in Neurosurgical Interventions for Patients with Mild Traumatic Brain Injury and Intracranial Hemorrhage
Source: Neurotrauma Rep. 2023 Mar 17;4(1):149–58. doi: 10.1089/neur.2022.0078 (PMC10024575; doi:10.1089/neur.2022.0078)
Supplement: Supplementary file 1 [file Supp_Data.odt]

| ICD10 Procedure | year | ICD9 Procedure |
| --- | --- | --- |
| 16070 | 2015 | 2.31 |
| 16071 | 2015 | 2.31 |
| 16072 | 2015 | 2.32 |
| 16073 | 2015 | 2.32 |
| 16074 | 2015 | 2.33 |
| 16075 | 2015 | 2.34 |
| 16076 | 2015 | 2.34 |
| 16077 | 2015 | 2.35 |
| 16078 | 2015 | 2.39 |
| 001607B | 2015 | 2.22 |
| 00160J0 | 2015 | 2.31 |
| 00160J1 | 2015 | 2.31 |
| 00160J2 | 2015 | 2.32 |
| 00160J3 | 2015 | 2.32 |
| 00160J4 | 2015 | 2.33 |
| 00160J5 | 2015 | 2.34 |
| 00160J6 | 2015 | 2.34 |
| 00160J7 | 2015 | 2.35 |
| 00160J8 | 2015 | 2.39 |
| 00160JB | 2015 | 2.22 |
| 00160K0 | 2015 | 2.31 |
| 00160K1 | 2015 | 2.31 |
| 00160K2 | 2015 | 2.32 |
| 00160K3 | 2015 | 2.32 |
| 00160K4 | 2015 | 2.33 |
| 00160K5 | 2015 | 2.34 |
| 00160K6 | 2015 | 2.34 |
| 00160K7 | 2015 | 2.35 |
| 00160K8 | 2015 | 2.39 |
| 00160KB | 2015 | 2.22 |
| 00160ZB | 2018 | 2.31 |
| 16370 | 2015 | 2.31 |
| 16371 | 2015 | 2.31 |
| 16372 | 2015 | 2.32 |
| 16373 | 2015 | 2.32 |
| 16374 | 2015 | 2.33 |
| 16375 | 2015 | 2.34 |
| 16376 | 2015 | 2.34 |
| 16377 | 2015 | 2.35 |
| 16378 | 2015 | 2.39 |
| 001637B | 2015 | 2.22 |
| 00163J0 | 2015 | 2.31 |
| 00163J1 | 2015 | 2.31 |
| 00163J2 | 2015 | 2.32 |
| 00163J3 | 2015 | 2.32 |
| 00163J4 | 2015 | 2.33 |
| 00163J5 | 2015 | 2.34 |
| 00163J6 | 2015 | 2.34 |
| 00163J7 | 2015 | 2.35 |
| 00163J8 | 2015 | 2.39 |
| 00163JB | 2015 | 2.22 |
| 00163K0 | 2015 | 2.31 |
| 00163K1 | 2015 | 2.31 |
| 00163K2 | 2015 | 2.32 |
| 00163K3 | 2015 | 2.32 |
| 00163K4 | 2015 | 2.33 |
| 00163K5 | 2015 | 2.34 |
| 00163K6 | 2015 | 2.34 |
| 00163K7 | 2015 | 2.35 |
| 00163K8 | 2015 | 2.39 |
| 00163KB | 2015 | 2.22 |
| 00163ZB | 2018 | 2.31 |
| 16470 | 2018 | 2.31 |
| 16471 | 2018 | 2.31 |
| 16472 | 2018 | 2.32 |
| 16473 | 2018 | 2.32 |
| 16474 | 2018 | 2.33 |
| 16475 | 2018 | 2.34 |
| 16476 | 2018 | 2.34 |
| 16477 | 2018 | 2.35 |
| 16478 | 2018 | 2.39 |
| 001647B | 2018 | 2.22 |
| 00164J0 | 2018 | 2.31 |
| 00164J1 | 2018 | 2.31 |
| 00164J2 | 2018 | 2.32 |
| 00164J3 | 2018 | 2.32 |
| 00164J4 | 2018 | 2.33 |
| 00164J5 | 2018 | 2.34 |
| 00164J6 | 2018 | 2.34 |
| 00164J7 | 2018 | 2.35 |
| 00164J8 | 2018 | 2.39 |
| 00164JB | 2018 | 2.22 |
| 00164K0 | 2018 | 2.31 |
| 00164K1 | 2018 | 2.31 |
| 00164K2 | 2018 | 2.32 |
| 00164K3 | 2018 | 2.32 |
| 00164K4 | 2018 | 2.33 |
| 00164K5 | 2018 | 2.34 |
| 00164K6 | 2018 | 2.34 |
| 00164K7 | 2018 | 2.35 |
| 00164K8 | 2018 | 2.39 |
| 00164KB | 2018 | 2.22 |
| 00164ZB | 2018 | 2.31 |
| 00500ZZ | 2015 | 1.59 |
| 00503ZZ | 2015 | 1.59 |
| 00504ZZ | 2015 | 1.59 |
| 00510ZZ | 2015 | 1.51 |
| 00513ZZ | 2015 | 1.51 |
| 00514ZZ | 2015 | 1.51 |
| 00520ZZ | 2015 | 1.51 |
| 00523ZZ | 2015 | 1.51 |
| 00524ZZ | 2015 | 1.51 |
| 00560ZZ | 2015 | 2.14 |
| 00563ZZ | 2015 | 2.14 |
| 00564ZZ | 2015 | 2.14 |
| 00570ZZ | 2015 | 1.59 |
| 00573ZZ | 2015 | 1.59 |
| 00574ZZ | 2015 | 1.59 |
| 00580ZZ | 2015 | 1.42 |
| 00583ZZ | 2015 | 1.42 |
| 00584ZZ | 2015 | 1.42 |
| 00590ZZ | 2015 | 1.41 |
| 00593ZZ | 2015 | 1.41 |
| 00594ZZ | 2015 | 1.41 |
| 005A0ZZ | 2015 | 1.41 |
| 005A3ZZ | 2015 | 1.41 |
| 005A4ZZ | 2015 | 1.41 |
| 005B0ZZ | 2015 | 1.59 |
| 005B3ZZ | 2015 | 1.59 |
| 005B4ZZ | 2015 | 1.59 |
| 005C0ZZ | 2015 | 1.59 |
| 005C3ZZ | 2015 | 1.59 |
| 005C4ZZ | 2015 | 1.59 |
| 005D0ZZ | 2015 | 1.59 |
| 005D3ZZ | 2015 | 1.59 |
| 005D4ZZ | 2015 | 1.59 |
| 00760ZZ | 2018 | 2.92 |
| 00763ZZ | 2018 | 2.92 |
| 00764ZZ | 2018 | 2.92 |
| 00800ZZ | 2015 | 1.32 |
| 00803ZZ | 2015 | 1.32 |
| 00804ZZ | 2015 | 1.32 |
| 00870ZZ | 2015 | 1.32 |
| 00873ZZ | 2015 | 1.32 |
| 00874ZZ | 2015 | 1.32 |
| 00880ZZ | 2015 | 1.42 |
| 00883ZZ | 2015 | 1.42 |
| 00884ZZ | 2015 | 1.42 |
| 009000Z | 2015 | 1.39 |
| 00900ZX | 2015 | 1.14 |
| 00900ZZ | 2015 | 1.39 |
| 009030Z | 2015 | 1.39 |
| 00903ZX | 2015 | 1.13 |
| 00903ZZ | 2015 | 1.39 |
| 009040Z | 2015 | 1.39 |
| 00904ZX | 2015 | 1.13 |
| 00904ZZ | 2015 | 1.39 |
| 009100Z | 2015 | 1.31 |
| 00910ZX | 2015 | 1.12 |
| 00910ZZ | 2015 | 1.31 |
| 009130Z | 2015 | 1.09 |
| 009130Z | 2017 | 2.22 |
| 00913ZX | 2015 | 1.11 |
| 00913ZZ | 2015 | 1.09 |
| 00913ZZ | 2017 | 2.22 |
| 009140Z | 2015 | 1.09 |
| 009140Z | 2017 | 2.22 |
| 00914ZX | 2015 | 1.11 |
| 00914ZZ | 2015 | 1.09 |
| 00914ZZ | 2017 | 2.22 |
| 009200Z | 2015 | 1.31 |
| 00920ZX | 2015 | 1.12 |
| 00920ZZ | 2015 | 1.31 |
| 009230Z | 2015 | 1.09 |
| 009230Z | 2017 | 2.22 |
| 00923ZX | 2015 | 1.11 |
| 00923ZZ | 2015 | 1.09 |
| 00923ZZ | 2017 | 2.22 |
| 009240Z | 2015 | 1.09 |
| 009240Z | 2017 | 2.22 |
| 00924ZX | 2015 | 1.11 |
| 00924ZZ | 2015 | 1.09 |
| 00924ZZ | 2017 | 2.22 |
| 009300Z | 2015 | 1.39 |
| 00930ZX | 2015 | 1.12 |
| 00930ZZ | 2015 | 1.39 |
| 009330Z | 2015 | 1.39 |
| 00933ZX | 2015 | 1.11 |
| 00933ZZ | 2015 | 1.39 |
| 009340Z | 2015 | 1.39 |
| 00934ZX | 2015 | 1.11 |
| 00934ZZ | 2015 | 1.39 |
| 009400Z | 2015 | 1.31 |
| 00940ZX | 2015 | 1.12 |
| 00940ZZ | 2015 | 1.31 |
| 009430Z | 2015 | 1.09 |
| 009430Z | 2017 | 2.22 |
| 00943ZX | 2015 | 1.11 |
| 00943ZZ | 2015 | 1.09 |
| 00943ZZ | 2017 | 2.22 |
| 009440Z | 2015 | 1.09 |
| 009440Z | 2017 | 2.22 |
| 00944ZX | 2015 | 1.11 |
| 00944ZZ | 2015 | 1.09 |
| 00944ZZ | 2017 | 2.22 |
| 009500Z | 2015 | 1.31 |
| 00950ZX | 2015 | 1.12 |
| 00950ZZ | 2015 | 1.31 |
| 009530Z | 2015 | 1.01 |
| 009530Z | 2017 | 2.22 |
| 00953ZX | 2015 | 1.11 |
| 00953ZZ | 2015 | 1.01 |
| 00953ZZ | 2017 | 2.22 |
| 009540Z | 2015 | 1.01 |
| 009540Z | 2017 | 2.22 |
| 00954ZX | 2015 | 1.11 |
| 00954ZZ | 2015 | 1.01 |
| 00954ZZ | 2017 | 2.22 |
| 009600Z | 2015 | 2.21 |
| 00960ZX | 2015 | 1.14 |
| 00960ZZ | 2015 | 1.39 |
| 009630Z | 2015 | 2.21 |
| 00963ZX | 2015 | 1.13 |
| 00963ZZ | 2015 | 1.09 |
| 00963ZZ | 2017 | 2.22 |
| 009640Z | 2015 | 2.21 |
| 00964ZX | 2015 | 1.13 |
| 00964ZZ | 2015 | 1.09 |
| 00964ZZ | 2017 | 2.22 |
| 009700Z | 2015 | 1.39 |
| 00970ZX | 2015 | 1.14 |
| 00970ZZ | 2015 | 1.39 |
| 009730Z | 2015 | 1.39 |
| 00973ZX | 2015 | 1.13 |
| 00973ZZ | 2015 | 1.39 |
| 009740Z | 2015 | 1.39 |
| 00974ZX | 2015 | 1.13 |
| 00974ZZ | 2015 | 1.39 |
| 009800Z | 2015 | 1.42 |
| 00980ZX | 2015 | 1.14 |
| 00980ZZ | 2015 | 1.42 |
| 009830Z | 2015 | 1.42 |
| 00983ZX | 2015 | 1.13 |
| 00983ZZ | 2015 | 1.42 |
| 009840Z | 2015 | 1.42 |
| 00984ZX | 2015 | 1.13 |
| 00984ZZ | 2015 | 1.42 |
| 009900Z | 2015 | 1.41 |
| 00990ZX | 2015 | 1.14 |
| 00990ZZ | 2015 | 1.41 |
| 009930Z | 2015 | 1.41 |
| 00993ZX | 2015 | 1.13 |
| 00993ZZ | 2015 | 1.41 |
| 009940Z | 2015 | 1.41 |
| 00994ZX | 2015 | 1.13 |
| 00994ZZ | 2015 | 1.41 |
| 009A00Z | 2015 | 1.41 |
| 009A0ZX | 2015 | 1.14 |
| 009A0ZZ | 2015 | 1.41 |
| 009A30Z | 2015 | 1.41 |
| 009A3ZX | 2015 | 1.13 |
| 009A3ZZ | 2015 | 1.41 |
| 009A40Z | 2015 | 1.41 |
| 009A4ZX | 2015 | 1.13 |
| 009A4ZZ | 2015 | 1.41 |
| 009B00Z | 2015 | 1.39 |
| 009B0ZX | 2015 | 1.14 |
| 009B0ZZ | 2015 | 1.39 |
| 009B30Z | 2015 | 1.39 |
| 009B3ZX | 2015 | 1.13 |
| 009B3ZZ | 2015 | 1.39 |
| 009B40Z | 2015 | 1.39 |
| 009B4ZX | 2015 | 1.13 |
| 009B4ZZ | 2015 | 1.39 |
| 009C00Z | 2015 | 1.39 |
| 009C0ZX | 2015 | 1.14 |
| 009C0ZZ | 2015 | 1.39 |
| 009C30Z | 2015 | 1.39 |
| 009C3ZX | 2015 | 1.13 |
| 009C3ZZ | 2015 | 1.39 |
| 009C40Z | 2015 | 1.39 |
| 009C4ZX | 2015 | 1.13 |
| 009C4ZZ | 2015 | 1.39 |
| 009D00Z | 2015 | 1.39 |
| 009D0ZX | 2015 | 1.14 |
| 009D0ZZ | 2015 | 1.39 |
| 009D30Z | 2015 | 1.39 |
| 009D3ZX | 2015 | 1.13 |
| 009D3ZZ | 2015 | 1.39 |
| 009D40Z | 2015 | 1.39 |
| 009D4ZX | 2015 | 1.13 |
| 009D4ZZ | 2015 | 1.39 |
| 00B00ZX | 2015 | 1.14 |
| 00B00ZZ | 2015 | 1.59 |
| 00B03ZX | 2015 | 1.13 |
| 00B03ZZ | 2015 | 1.59 |
| 00B04ZX | 2015 | 1.13 |
| 00B04ZZ | 2015 | 1.59 |
| 00B10ZX | 2015 | 1.12 |
| 00B10ZZ | 2015 | 1.51 |
| 00B13ZX | 2015 | 1.11 |
| 00B13ZZ | 2015 | 1.51 |
| 00B14ZX | 2015 | 1.11 |
| 00B14ZZ | 2015 | 1.51 |
| 00B20ZX | 2015 | 1.12 |
| 00B20ZZ | 2015 | 1.51 |
| 00B23ZX | 2015 | 1.11 |
| 00B23ZZ | 2015 | 1.51 |
| 00B24ZX | 2015 | 1.11 |
| 00B24ZZ | 2015 | 1.51 |
| 00B60ZX | 2015 | 1.14 |
| 00B60ZZ | 2015 | 1.59 |
| 00B63ZX | 2015 | 1.13 |
| 00B63ZZ | 2015 | 1.59 |
| 00B64ZX | 2015 | 1.13 |
| 00B64ZZ | 2015 | 1.59 |
| 00B70ZX | 2015 | 1.14 |
| 00B70ZZ | 2015 | 1.53 |
| 00B70ZZ | 2015 | 1.59 |
| 00B73ZX | 2015 | 1.13 |
| 00B73ZZ | 2015 | 1.53 |
| 00B73ZZ | 2015 | 1.59 |
| 00B74ZX | 2015 | 1.13 |
| 00B74ZZ | 2015 | 1.53 |
| 00B74ZZ | 2015 | 1.59 |
| 00B80ZX | 2015 | 1.14 |
| 00B80ZZ | 2015 | 1.42 |
| 00B83ZX | 2015 | 1.13 |
| 00B83ZZ | 2015 | 1.42 |
| 00B84ZX | 2015 | 1.13 |
| 00B84ZZ | 2015 | 1.42 |
| 00B90ZX | 2015 | 1.14 |
| 00B90ZZ | 2015 | 1.41 |
| 00B93ZX | 2015 | 1.13 |
| 00B93ZZ | 2015 | 1.41 |
| 00B94ZX | 2015 | 1.13 |
| 00B94ZZ | 2015 | 1.41 |
| 00BA0ZX | 2015 | 1.14 |
| 00BA0ZZ | 2015 | 1.41 |
| 00BA3ZX | 2015 | 1.13 |
| 00BA3ZZ | 2015 | 1.41 |
| 00BA4ZX | 2015 | 1.13 |
| 00BA4ZZ | 2015 | 1.41 |
| 00BB0ZX | 2015 | 1.14 |
| 00BB0ZZ | 2015 | 1.59 |
| 00BB3ZX | 2015 | 1.13 |
| 00BB3ZZ | 2015 | 1.59 |
| 00BB4ZX | 2015 | 1.13 |
| 00BB4ZZ | 2015 | 1.59 |
| 00BC0ZX | 2015 | 1.14 |
| 00BC0ZZ | 2015 | 1.59 |
| 00BC3ZX | 2015 | 1.13 |
| 00BC3ZZ | 2015 | 1.59 |
| 00BC4ZX | 2015 | 1.13 |
| 00BC4ZZ | 2015 | 1.59 |
| 00BD0ZX | 2015 | 1.14 |
| 00BD0ZZ | 2015 | 1.59 |
| 00BD3ZX | 2015 | 1.13 |
| 00BD3ZZ | 2015 | 1.59 |
| 00BD4ZX | 2015 | 1.13 |
| 00BD4ZZ | 2015 | 1.59 |
| 00C00ZZ | 2015 | 1.39 |
| 00C03ZZ | 2015 | 1.39 |
| 00C04ZZ | 2015 | 1.39 |
| 00C10ZZ | 2015 | 1.31 |
| 00C13ZZ | 2015 | 1.31 |
| 00C14ZZ | 2015 | 1.31 |
| 00C20ZZ | 2015 | 1.31 |
| 00C23ZZ | 2015 | 1.31 |
| 00C24ZZ | 2015 | 1.31 |
| 00C30ZZ | 2015 | 1.39 |
| 00C33ZZ | 2015 | 1.39 |
| 00C34ZZ | 2015 | 1.39 |
| 00C40ZZ | 2015 | 1.31 |
| 00C43ZZ | 2015 | 1.31 |
| 00C44ZZ | 2015 | 1.31 |
| 00C50ZZ | 2015 | 1.31 |
| 00C53ZZ | 2015 | 1.31 |
| 00C54ZZ | 2015 | 1.31 |
| 00C60ZZ | 2015 | 1.39 |
| 00C63ZZ | 2015 | 1.39 |
| 00C64ZZ | 2015 | 1.39 |
| 00C70ZZ | 2015 | 1.39 |
| 00C73ZZ | 2015 | 1.39 |
| 00C74ZZ | 2015 | 1.39 |
| 00C80ZZ | 2015 | 1.42 |
| 00C83ZZ | 2015 | 1.42 |
| 00C84ZZ | 2015 | 1.42 |
| 00C90ZZ | 2015 | 1.41 |
| 00C93ZZ | 2015 | 1.41 |
| 00C94ZZ | 2015 | 1.41 |
| 00CA0ZZ | 2015 | 1.41 |
| 00CA3ZZ | 2015 | 1.41 |
| 00CA4ZZ | 2015 | 1.41 |
| 00CB0ZZ | 2015 | 1.39 |
| 00CB3ZZ | 2015 | 1.39 |
| 00CB4ZZ | 2015 | 1.39 |
| 00CC0ZZ | 2015 | 1.39 |
| 00CC3ZZ | 2015 | 1.39 |
| 00CC4ZZ | 2015 | 1.39 |
| 00CD0ZZ | 2015 | 1.39 |
| 00CD3ZZ | 2015 | 1.39 |
| 00CD4ZZ | 2015 | 1.39 |
| 00D10ZZ | 2015 | 1.51 |
| 00D13ZZ | 2015 | 1.51 |
| 00D14ZZ | 2015 | 1.51 |
| 00D20ZZ | 2015 | 1.51 |
| 00D23ZZ | 2015 | 1.51 |
| 00D24ZZ | 2015 | 1.51 |
| 00F30ZZ | 2015 | 2.92 |
| 00F33ZZ | 2015 | 2.92 |
| 00F34ZZ | 2015 | 2.92 |
| 00F40ZZ | 2015 | 2.92 |
| 00F43ZZ | 2015 | 2.92 |
| 00F44ZZ | 2015 | 2.92 |
| 00F50ZZ | 2015 | 2.92 |
| 00F53ZZ | 2015 | 2.92 |
| 00F54ZZ | 2015 | 2.92 |
| 00F60ZZ | 2015 | 2.92 |
| 00F63ZZ | 2015 | 2.92 |
| 00F64ZZ | 2015 | 2.92 |
| 00H002Z | 2015 | 1.39 |
| 00H003Z | 2015 | 1.39 |
| 00H00MZ | 2015 | 2.93 |
| 00H00MZ | 2015 | 2.96 |
| 00H00YZ | 2018 | 1.39 |
| 00H032Z | 2015 | 1.39 |
| 00H033Z | 2015 | 1.28 |
| 00H03MZ | 2015 | 2.93 |
| 00H03MZ | 2015 | 2.96 |
| 00H03YZ | 2018 | 1.39 |
| 00H042Z | 2015 | 1.39 |
| 00H043Z | 2015 | 1.39 |
| 00H04MZ | 2015 | 2.93 |
| 00H04MZ | 2015 | 2.96 |
| 00H04YZ | 2018 | 1.39 |
| 00H602Z | 2015 | 1.39 |
| 00H603Z | 2015 | 1.39 |
| 00H60MZ | 2015 | 2.93 |
| 00H60YZ | 2018 | 1.39 |
| 00H632Z | 2015 | 1.39 |
| 00H633Z | 2015 | 1.39 |
| 00H63MZ | 2015 | 2.93 |
| 00H63YZ | 2018 | 1.39 |
| 00H642Z | 2015 | 1.39 |
| 00H643Z | 2015 | 1.39 |
| 00H64MZ | 2015 | 2.93 |
| 00H64YZ | 2018 | 1.39 |
| 00J00ZZ | 2015 | 1.24 |
| 00J03ZZ | 2015 | 1.18 |
| 00J04ZZ | 2015 | 1.18 |
| 00K00ZZ | 2015 | 1.18 |
| 00K03ZZ | 2015 | 1.18 |
| 00K04ZZ | 2015 | 1.18 |
| 00K70ZZ | 2015 | 1.18 |
| 00K73ZZ | 2015 | 1.18 |
| 00K74ZZ | 2015 | 1.18 |
| 00K80ZZ | 2015 | 1.18 |
| 00K83ZZ | 2015 | 1.18 |
| 00K84ZZ | 2015 | 1.18 |
| 00K90ZZ | 2015 | 1.18 |
| 00K93ZZ | 2015 | 1.18 |
| 00K94ZZ | 2015 | 1.18 |
| 00KA0ZZ | 2015 | 1.18 |
| 00KA3ZZ | 2015 | 1.18 |
| 00KA4ZZ | 2015 | 1.18 |
| 00KB0ZZ | 2015 | 1.18 |
| 00KB3ZZ | 2015 | 1.18 |
| 00KB4ZZ | 2015 | 1.18 |
| 00KC0ZZ | 2015 | 1.18 |
| 00KC3ZZ | 2015 | 1.18 |
| 00KC4ZZ | 2015 | 1.18 |
| 00KD0ZZ | 2015 | 1.18 |
| 00KD3ZZ | 2015 | 1.18 |
| 00KD4ZZ | 2015 | 1.18 |
| 00N00ZZ | 2015 | 2.91 |
| 00N03ZZ | 2015 | 2.91 |
| 00N04ZZ | 2015 | 2.91 |
| 00N10ZZ | 2015 | 2.91 |
| 00N13ZZ | 2015 | 2.91 |
| 00N14ZZ | 2015 | 2.91 |
| 00N20ZZ | 2015 | 2.91 |
| 00N23ZZ | 2015 | 2.91 |
| 00N24ZZ | 2015 | 2.91 |
| 00N60ZZ | 2015 | 2.92 |
| 00N63ZZ | 2015 | 2.92 |
| 00N64ZZ | 2015 | 2.92 |
| 00N70ZZ | 2015 | 2.91 |
| 00N73ZZ | 2015 | 2.91 |
| 00N74ZZ | 2015 | 2.91 |
| 00N80ZZ | 2015 | 2.92 |
| 00N83ZZ | 2015 | 2.92 |
| 00N84ZZ | 2015 | 2.92 |
| 00N90ZZ | 2015 | 1.41 |
| 00N93ZZ | 2015 | 1.41 |
| 00N94ZZ | 2015 | 1.41 |
| 00NA0ZZ | 2015 | 1.41 |
| 00NA3ZZ | 2015 | 1.41 |
| 00NA4ZZ | 2015 | 1.41 |
| 00NB0ZZ | 2015 | 2.92 |
| 00NB3ZZ | 2015 | 2.92 |
| 00NB4ZZ | 2015 | 2.92 |
| 00NC0ZZ | 2015 | 2.92 |
| 00NC3ZZ | 2015 | 2.92 |
| 00NC4ZZ | 2015 | 2.92 |
| 00ND0ZZ | 2015 | 2.92 |
| 00ND3ZZ | 2015 | 2.92 |
| 00ND4ZZ | 2015 | 2.92 |
| 00P000Z | 2015 | 1.39 |
| 00P002Z | 2015 | 1.39 |
| 00P003Z | 2015 | 1.39 |
| 00P007Z | 2015 | 1.39 |
| 00P00JZ | 2015 | 1.39 |
| 00P00KZ | 2015 | 1.39 |
| 00P00MZ | 2015 | 1.22 |
| 00P00YZ | 2018 | 1.39 |
| 00P030Z | 2015 | 1.39 |
| 00P032Z | 2015 | 1.39 |
| 00P033Z | 2015 | 1.39 |
| 00P037Z | 2015 | 1.39 |
| 00P03JZ | 2015 | 1.39 |
| 00P03KZ | 2015 | 1.39 |
| 00P03MZ | 2015 | 1.22 |
| 00P03YZ | 2018 | 1.39 |
| 00P040Z | 2015 | 1.39 |
| 00P042Z | 2015 | 1.39 |
| 00P043Z | 2015 | 1.39 |
| 00P047Z | 2015 | 1.39 |
| 00P04JZ | 2015 | 1.39 |
| 00P04KZ | 2015 | 1.39 |
| 00P04MZ | 2015 | 1.22 |
| 00P04YZ | 2018 | 1.39 |
| 00P600Z | 2015 | 1.39 |
| 00P602Z | 2015 | 1.39 |
| 00P603Z | 2015 | 1.39 |
| 00P60JZ | 2015 | 2.43 |
| 00P60MZ | 2015 | 1.22 |
| 00P60YZ | 2018 | 1.39 |
| 00P630Z | 2015 | 1.39 |
| 00P632Z | 2015 | 1.39 |
| 00P633Z | 2015 | 1.39 |
| 00P63JZ | 2015 | 2.43 |
| 00P63MZ | 2015 | 1.22 |
| 00P63YZ | 2018 | 1.39 |
| 00P640Z | 2015 | 1.39 |
| 00P642Z | 2015 | 1.39 |
| 00P643Z | 2015 | 1.39 |
| 00P64JZ | 2015 | 2.43 |
| 00P64MZ | 2015 | 1.22 |
| 00P64YZ | 2018 | 1.39 |
| 00P6X2Z | 2015 | 1.39 |
| 00P6XMZ | 2015 | 1.22 |
| 00Q00ZZ | 2015 | 2.92 |
| 00Q03ZZ | 2015 | 2.92 |
| 00Q04ZZ | 2015 | 2.92 |
| 00Q10ZZ | 2015 | 2.12 |
| 00Q13ZZ | 2015 | 2.12 |
| 00Q14ZZ | 2015 | 2.12 |
| 00Q20ZZ | 2015 | 2.11 |
| 00Q23ZZ | 2015 | 2.11 |
| 00Q24ZZ | 2015 | 2.11 |
| 00Q60ZZ | 2015 | 2.92 |
| 00Q63ZZ | 2015 | 2.92 |
| 00Q64ZZ | 2015 | 2.92 |
| 00Q70ZZ | 2015 | 2.92 |
| 00Q73ZZ | 2015 | 2.92 |
| 00Q74ZZ | 2015 | 2.92 |
| 00Q80ZZ | 2015 | 2.92 |
| 00Q83ZZ | 2015 | 2.92 |
| 00Q84ZZ | 2015 | 2.92 |
| 00Q90ZZ | 2015 | 1.41 |
| 00Q93ZZ | 2015 | 1.41 |
| 00Q94ZZ | 2015 | 1.41 |
| 00QA0ZZ | 2015 | 1.41 |
| 00QA3ZZ | 2015 | 1.41 |
| 00QA4ZZ | 2015 | 1.41 |
| 00QB0ZZ | 2015 | 2.92 |
| 00QB3ZZ | 2015 | 2.92 |
| 00QB4ZZ | 2015 | 2.92 |
| 00QC0ZZ | 2015 | 2.92 |
| 00QC3ZZ | 2015 | 2.92 |
| 00QC4ZZ | 2015 | 2.92 |
| 00QD0ZZ | 2015 | 2.92 |
| 00QD3ZZ | 2015 | 2.92 |
| 00QD4ZZ | 2015 | 2.92 |
| 00R107Z | 2018 | 2.12 |
| 00R10JZ | 2018 | 2.12 |
| 00R10KZ | 2018 | 2.12 |
| 00R147Z | 2018 | 2.12 |
| 00R14JZ | 2018 | 2.12 |
| 00R14KZ | 2018 | 2.12 |
| 00R207Z | 2018 | 2.12 |
| 00R20JZ | 2018 | 2.12 |
| 00R20KZ | 2018 | 2.12 |
| 00R247Z | 2018 | 2.12 |
| 00R24JZ | 2018 | 2.12 |
| 00R24KZ | 2018 | 2.12 |
| 00R607Z | 2018 | 2.92 |
| 00R60JZ | 2018 | 2.92 |
| 00R60KZ | 2018 | 2.92 |
| 00R647Z | 2018 | 2.92 |
| 00R64JZ | 2018 | 2.92 |
| 00R64KZ | 2018 | 2.92 |
| 00T70ZZ | 2015 | 1.52 |
| 00T73ZZ | 2015 | 1.52 |
| 00T74ZZ | 2015 | 1.52 |
| 00U107Z | 2015 | 2.12 |
| 00U10JZ | 2015 | 2.12 |
| 00U10KZ | 2015 | 2.12 |
| 00U137Z | 2015 | 2.12 |
| 00U13JZ | 2015 | 2.12 |
| 00U13KZ | 2015 | 2.12 |
| 00U147Z | 2015 | 2.12 |
| 00U14JZ | 2015 | 2.12 |
| 00U14KZ | 2015 | 2.12 |
| 00U207Z | 2015 | 2.12 |
| 00U20JZ | 2015 | 2.12 |
| 00U20KZ | 2015 | 2.12 |
| 00U237Z | 2015 | 2.12 |
| 00U23JZ | 2015 | 2.12 |
| 00U23KZ | 2015 | 2.12 |
| 00U247Z | 2015 | 2.12 |
| 00U24JZ | 2015 | 2.12 |
| 00U24KZ | 2015 | 2.12 |
| 00U607Z | 2018 | 2.92 |
| 00U60JZ | 2018 | 2.92 |
| 00U60KZ | 2018 | 2.92 |
| 00U637Z | 2018 | 2.92 |
| 00U63JZ | 2018 | 2.92 |
| 00U63KZ | 2018 | 2.92 |
| 00U647Z | 2018 | 2.92 |
| 00U64JZ | 2018 | 2.92 |
| 00U64KZ | 2018 | 2.92 |
| 00W000Z | 2015 | 1.39 |
| 00W002Z | 2015 | 1.39 |
| 00W003Z | 2015 | 1.39 |
| 00W007Z | 2015 | 1.39 |
| 00W00JZ | 2015 | 1.24 |
| 00W00KZ | 2015 | 1.24 |
| 00W00MZ | 2015 | 1.39 |
| 00W00YZ | 2018 | 1.39 |
| 00W030Z | 2015 | 1.39 |
| 00W032Z | 2015 | 1.39 |
| 00W033Z | 2015 | 1.39 |
| 00W037Z | 2015 | 1.39 |
| 00W03JZ | 2015 | 1.39 |
| 00W03KZ | 2015 | 1.39 |
| 00W03MZ | 2015 | 1.39 |
| 00W03YZ | 2018 | 1.39 |
| 00W040Z | 2015 | 1.39 |
| 00W042Z | 2015 | 1.39 |
| 00W043Z | 2015 | 1.39 |
| 00W047Z | 2015 | 1.39 |
| 00W04JZ | 2015 | 1.39 |
| 00W04KZ | 2015 | 1.39 |
| 00W04MZ | 2015 | 1.39 |
| 00W04YZ | 2018 | 1.39 |
| 00W600Z | 2015 | 1.39 |
| 00W602Z | 2015 | 1.39 |
| 00W603Z | 2015 | 1.39 |
| 00W60JZ | 2015 | 2.42 |
| 00W60MZ | 2015 | 1.39 |
| 00W60YZ | 2018 | 1.39 |
| 00W630Z | 2015 | 1.39 |
| 00W632Z | 2015 | 1.39 |
| 00W633Z | 2015 | 1.39 |
| 00W63JZ | 2015 | 2.42 |
| 00W63MZ | 2015 | 1.39 |
| 00W63YZ | 2018 | 1.39 |
| 00W640Z | 2015 | 1.39 |
| 00W642Z | 2015 | 1.39 |
| 00W643Z | 2015 | 1.39 |
| 00W64JZ | 2015 | 2.42 |
| 00W64MZ | 2015 | 1.39 |
| 00W64YZ | 2018 | 1.39 |
| 03LG0CZ | 2015 | 2.13 |
| 03LG0ZZ | 2015 | 2.13 |
| 03LG3CZ | 2015 | 2.13 |
| 03LG3ZZ | 2015 | 2.13 |
| 03LG4CZ | 2015 | 2.13 |
| 03LG4ZZ | 2015 | 2.13 |
| 0N500ZZ | 2015 | 1.25 |
| 0N503ZZ | 2015 | 1.25 |
| 0N504ZZ | 2015 | 1.25 |
| 0N510ZZ | 2015 | 1.25 |
| 0N513ZZ | 2015 | 1.25 |
| 0N514ZZ | 2015 | 1.25 |
| 0N520ZZ | 2015 | 1.25 |
| 0N523ZZ | 2015 | 1.25 |
| 0N524ZZ | 2015 | 1.25 |
| 0N530ZZ | 2015 | 1.25 |
| 0N533ZZ | 2015 | 1.25 |
| 0N534ZZ | 2015 | 1.25 |
| 0N540ZZ | 2015 | 1.25 |
| 0N543ZZ | 2015 | 1.25 |
| 0N544ZZ | 2015 | 1.25 |
| 0N550ZZ | 2015 | 1.25 |
| 0N553ZZ | 2015 | 1.25 |
| 0N554ZZ | 2015 | 1.25 |
| 0N560ZZ | 2015 | 1.25 |
| 0N563ZZ | 2015 | 1.25 |
| 0N564ZZ | 2015 | 1.25 |
| 0N570ZZ | 2015 | 1.25 |
| 0N573ZZ | 2015 | 1.25 |
| 0N574ZZ | 2015 | 1.25 |
| 0N580ZZ | 2015 | 1.25 |
| 0N583ZZ | 2015 | 1.25 |
| 0N584ZZ | 2015 | 1.25 |
| 0N800ZZ | 2015 | 1.24 |
| 0N803ZZ | 2015 | 1.24 |
| 0N804ZZ | 2015 | 1.24 |
| 0N810ZZ | 2015 | 1.24 |
| 0N813ZZ | 2015 | 1.24 |
| 0N814ZZ | 2015 | 1.24 |
| 0N820ZZ | 2015 | 1.24 |
| 0N823ZZ | 2015 | 1.24 |
| 0N824ZZ | 2015 | 1.24 |
| 0N830ZZ | 2015 | 1.24 |
| 0N833ZZ | 2015 | 1.24 |
| 0N834ZZ | 2015 | 1.24 |
| 0N840ZZ | 2015 | 1.24 |
| 0N843ZZ | 2015 | 1.24 |
| 0N844ZZ | 2015 | 1.24 |
| 0N850ZZ | 2015 | 1.24 |
| 0N853ZZ | 2015 | 1.24 |
| 0N854ZZ | 2015 | 1.24 |
| 0N860ZZ | 2015 | 1.24 |
| 0N863ZZ | 2015 | 1.24 |
| 0N864ZZ | 2015 | 1.24 |
| 0N870ZZ | 2015 | 1.24 |
| 0N873ZZ | 2015 | 1.24 |
| 0N874ZZ | 2015 | 1.24 |
| 0N880ZZ | 2015 | 1.24 |
| 0N883ZZ | 2015 | 1.24 |
| 0N884ZZ | 2015 | 1.24 |
| 0N9000Z | 2015 | 1.21 |
| 0N900ZX | 2015 | 1.15 |
| 0N900ZZ | 2015 | 1.21 |
| 0N9030Z | 2017 | 1.09 |
| 0N9030Z | 2015 | 1.21 |
| 0N903ZX | 2015 | 1.15 |
| 0N903ZZ | 2017 | 1.09 |
| 0N903ZZ | 2015 | 1.21 |
| 0N9040Z | 2015 | 1.21 |
| 0N904ZX | 2015 | 1.15 |
| 0N904ZZ | 2015 | 1.21 |
| 0N9100Z | 2015 | 1.21 |
| 0N910ZX | 2015 | 1.15 |
| 0N910ZZ | 2015 | 1.21 |
| 0N9130Z | 2017 | 1.09 |
| 0N9130Z | 2015 | 1.21 |
| 0N913ZX | 2015 | 1.15 |
| 0N913ZZ | 2017 | 1.09 |
| 0N913ZZ | 2015 | 1.21 |
| 0N9140Z | 2015 | 1.21 |
| 0N914ZX | 2015 | 1.15 |
| 0N914ZZ | 2015 | 1.21 |
| 0N9200Z | 2015 | 1.21 |
| 0N920ZX | 2015 | 1.15 |
| 0N920ZZ | 2015 | 1.21 |
| 0N9230Z | 2017 | 1.09 |
| 0N9230Z | 2015 | 1.21 |
| 0N923ZX | 2015 | 1.15 |
| 0N923ZZ | 2017 | 1.09 |
| 0N923ZZ | 2015 | 1.21 |
| 0N9240Z | 2015 | 1.21 |
| 0N924ZX | 2015 | 1.15 |
| 0N924ZZ | 2015 | 1.21 |
| 0N9300Z | 2015 | 1.21 |
| 0N930ZX | 2015 | 1.15 |
| 0N930ZZ | 2015 | 1.21 |
| 0N9330Z | 2017 | 1.09 |
| 0N9330Z | 2015 | 1.21 |
| 0N933ZX | 2015 | 1.15 |
| 0N933ZZ | 2017 | 1.09 |
| 0N933ZZ | 2015 | 1.21 |
| 0N9340Z | 2015 | 1.21 |
| 0N934ZX | 2015 | 1.15 |
| 0N934ZZ | 2015 | 1.21 |
| 0N9400Z | 2015 | 1.21 |
| 0N940ZX | 2015 | 1.15 |
| 0N940ZZ | 2015 | 1.21 |
| 0N9430Z | 2017 | 1.09 |
| 0N9430Z | 2015 | 1.21 |
| 0N943ZX | 2015 | 1.15 |
| 0N943ZZ | 2017 | 1.09 |
| 0N943ZZ | 2015 | 1.21 |
| 0N9440Z | 2015 | 1.21 |
| 0N944ZX | 2015 | 1.15 |
| 0N944ZZ | 2015 | 1.21 |
| 0N9500Z | 2015 | 1.21 |
| 0N950ZX | 2015 | 1.15 |
| 0N950ZZ | 2015 | 1.21 |
| 0N9530Z | 2017 | 1.09 |
| 0N9530Z | 2015 | 1.21 |
| 0N953ZX | 2015 | 1.15 |
| 0N953ZZ | 2017 | 1.09 |
| 0N953ZZ | 2015 | 1.21 |
| 0N9540Z | 2015 | 1.21 |
| 0N954ZX | 2015 | 1.15 |
| 0N954ZZ | 2015 | 1.21 |
| 0N9600Z | 2015 | 1.21 |
| 0N960ZX | 2015 | 1.15 |
| 0N960ZZ | 2015 | 1.21 |
| 0N9630Z | 2017 | 1.09 |
| 0N9630Z | 2015 | 1.21 |
| 0N963ZX | 2015 | 1.15 |
| 0N963ZZ | 2017 | 1.09 |
| 0N963ZZ | 2015 | 1.21 |
| 0N9640Z | 2015 | 1.21 |
| 0N964ZX | 2015 | 1.15 |
| 0N964ZZ | 2015 | 1.21 |
| 0N9700Z | 2015 | 1.21 |
| 0N970ZX | 2015 | 1.15 |
| 0N970ZZ | 2015 | 1.21 |
| 0N9730Z | 2017 | 1.09 |
| 0N9730Z | 2015 | 1.21 |
| 0N973ZX | 2015 | 1.15 |
| 0N973ZZ | 2017 | 1.09 |
| 0N973ZZ | 2015 | 1.21 |
| 0N9740Z | 2015 | 1.21 |
| 0N974ZX | 2015 | 1.15 |
| 0N974ZZ | 2015 | 1.21 |
| 0N9800Z | 2015 | 1.21 |
| 0N980ZX | 2015 | 1.15 |
| 0N980ZZ | 2015 | 1.21 |
| 0N9830Z | 2017 | 1.09 |
| 0N9830Z | 2015 | 1.21 |
| 0N983ZX | 2015 | 1.15 |
| 0N983ZZ | 2017 | 1.09 |
| 0N983ZZ | 2015 | 1.21 |
| 0N9840Z | 2015 | 1.21 |
| 0N984ZX | 2015 | 1.15 |
| 0N984ZZ | 2015 | 1.21 |
| 0N9C30Z | 2017 | 1.09 |
| 0N9C3ZZ | 2017 | 1.09 |
| 0N9D30Z | 2017 | 1.09 |
| 0N9D3ZZ | 2017 | 1.09 |
| 0N9F30Z | 2017 | 1.09 |
| 0N9F3ZZ | 2017 | 1.09 |
| 0N9G30Z | 2017 | 1.09 |
| 0N9G3ZZ | 2017 | 1.09 |
| 0N9H30Z | 2017 | 1.09 |
| 0N9H3ZZ | 2017 | 1.09 |
| 0N9J30Z | 2017 | 1.09 |
| 0N9J3ZZ | 2017 | 1.09 |
| 0N9K30Z | 2017 | 1.09 |
| 0N9K3ZZ | 2017 | 1.09 |
| 0N9L30Z | 2017 | 1.09 |
| 0N9L3ZZ | 2017 | 1.09 |
| 0N9M30Z | 2017 | 1.09 |
| 0N9M3ZZ | 2017 | 1.09 |
| 0N9N30Z | 2017 | 1.09 |
| 0N9N3ZZ | 2017 | 1.09 |
| 0N9P30Z | 2017 | 1.09 |
| 0N9P3ZZ | 2017 | 1.09 |
| 0N9Q30Z | 2017 | 1.09 |
| 0N9Q3ZZ | 2017 | 1.09 |
| 0N9X30Z | 2017 | 1.09 |
| 0N9X3ZZ | 2017 | 1.09 |
| 0NB00ZX | 2015 | 1.15 |
| 0NB00ZZ | 2015 | 1.25 |
| 0NB00ZZ | 2015 | 1.6 |
| 0NB03ZX | 2015 | 1.15 |
| 0NB03ZZ | 2015 | 1.25 |
| 0NB03ZZ | 2015 | 1.6 |
| 0NB04ZX | 2015 | 1.15 |
| 0NB04ZZ | 2015 | 1.25 |
| 0NB04ZZ | 2015 | 1.6 |
| 0NB10ZX | 2015 | 1.15 |
| 0NB10ZZ | 2015 | 1.25 |
| 0NB10ZZ | 2015 | 1.6 |
| 0NB13ZX | 2015 | 1.15 |
| 0NB13ZZ | 2015 | 1.25 |
| 0NB13ZZ | 2015 | 1.6 |
| 0NB14ZX | 2015 | 1.15 |
| 0NB14ZZ | 2015 | 1.25 |
| 0NB14ZZ | 2015 | 1.6 |
| 0NB20ZX | 2015 | 1.15 |
| 0NB20ZZ | 2015 | 1.25 |
| 0NB20ZZ | 2015 | 1.6 |
| 0NB23ZX | 2015 | 1.15 |
| 0NB23ZZ | 2015 | 1.25 |
| 0NB23ZZ | 2015 | 1.6 |
| 0NB24ZX | 2015 | 1.15 |
| 0NB24ZZ | 2015 | 1.25 |
| 0NB24ZZ | 2015 | 1.6 |
| 0NB30ZX | 2015 | 1.15 |
| 0NB30ZZ | 2015 | 1.25 |
| 0NB30ZZ | 2015 | 1.6 |
| 0NB33ZX | 2015 | 1.15 |
| 0NB33ZZ | 2015 | 1.25 |
| 0NB33ZZ | 2015 | 1.6 |
| 0NB34ZX | 2015 | 1.15 |
| 0NB34ZZ | 2015 | 1.25 |
| 0NB34ZZ | 2015 | 1.6 |
| 0NB40ZX | 2015 | 1.15 |
| 0NB40ZZ | 2015 | 1.25 |
| 0NB40ZZ | 2015 | 1.6 |
| 0NB43ZX | 2015 | 1.15 |
| 0NB43ZZ | 2015 | 1.25 |
| 0NB43ZZ | 2015 | 1.6 |
| 0NB44ZX | 2015 | 1.15 |
| 0NB44ZZ | 2015 | 1.25 |
| 0NB44ZZ | 2015 | 1.6 |
| 0NB50ZX | 2015 | 1.15 |
| 0NB50ZZ | 2015 | 1.25 |
| 0NB50ZZ | 2015 | 1.6 |
| 0NB53ZX | 2015 | 1.15 |
| 0NB53ZZ | 2015 | 1.25 |
| 0NB53ZZ | 2015 | 1.6 |
| 0NB54ZX | 2015 | 1.15 |
| 0NB54ZZ | 2015 | 1.25 |
| 0NB54ZZ | 2015 | 1.6 |
| 0NB60ZX | 2015 | 1.15 |
| 0NB60ZZ | 2015 | 1.25 |
| 0NB60ZZ | 2015 | 1.6 |
| 0NB63ZX | 2015 | 1.15 |
| 0NB63ZZ | 2015 | 1.25 |
| 0NB63ZZ | 2015 | 1.6 |
| 0NB64ZX | 2015 | 1.15 |
| 0NB64ZZ | 2015 | 1.25 |
| 0NB64ZZ | 2015 | 1.6 |
| 0NB70ZX | 2015 | 1.15 |
| 0NB70ZZ | 2015 | 1.25 |
| 0NB70ZZ | 2015 | 1.6 |
| 0NB73ZX | 2015 | 1.15 |
| 0NB73ZZ | 2015 | 1.25 |
| 0NB73ZZ | 2015 | 1.6 |
| 0NB74ZX | 2015 | 1.15 |
| 0NB74ZZ | 2015 | 1.25 |
| 0NB74ZZ | 2015 | 1.6 |
| 0NB80ZX | 2015 | 1.15 |
| 0NB80ZZ | 2015 | 1.25 |
| 0NB80ZZ | 2015 | 1.6 |
| 0NB83ZX | 2015 | 1.15 |
| 0NB83ZZ | 2015 | 1.25 |
| 0NB83ZZ | 2015 | 1.6 |
| 0NB84ZX | 2015 | 1.15 |
| 0NB84ZZ | 2015 | 1.25 |
| 0NB84ZZ | 2015 | 1.6 |
| 0NC10ZZ | 2015 | 1.24 |
| 0NC13ZZ | 2015 | 1.24 |
| 0NC14ZZ | 2015 | 1.24 |
| 0NC20ZZ | 2015 | 1.24 |
| 0NC23ZZ | 2015 | 1.24 |
| 0NC24ZZ | 2015 | 1.24 |
| 0NC30ZZ | 2015 | 1.24 |
| 0NC33ZZ | 2015 | 1.24 |
| 0NC34ZZ | 2015 | 1.24 |
| 0NC40ZZ | 2015 | 1.24 |
| 0NC43ZZ | 2015 | 1.24 |
| 0NC44ZZ | 2015 | 1.24 |
| 0NC50ZZ | 2015 | 1.24 |
| 0NC53ZZ | 2015 | 1.24 |
| 0NC54ZZ | 2015 | 1.24 |
| 0NC60ZZ | 2015 | 1.24 |
| 0NC63ZZ | 2015 | 1.24 |
| 0NC64ZZ | 2015 | 1.24 |
| 0NC70ZZ | 2015 | 1.24 |
| 0NC73ZZ | 2015 | 1.24 |
| 0NC74ZZ | 2015 | 1.24 |
| 0NC80ZZ | 2015 | 1.24 |
| 0NC83ZZ | 2015 | 1.24 |
| 0NC84ZZ | 2015 | 1.24 |
| 0NH004Z | 2015 | 2.94 |
| 0NH00MZ | 2015 | 1.24 |
| 0NH00NZ | 2015 | 1.2 |
| 0NH034Z | 2015 | 2.94 |
| 0NH03MZ | 2015 | 1.24 |
| 0NH044Z | 2015 | 2.94 |
| 0NH04MZ | 2015 | 1.24 |
| 0NH104Z | 2015 | 2.06 |
| 0NH134Z | 2015 | 2.06 |
| 0NH144Z | 2015 | 2.06 |
| 0NH204Z | 2015 | 2.06 |
| 0NH234Z | 2015 | 2.06 |
| 0NH244Z | 2015 | 2.06 |
| 0NH304Z | 2015 | 2.06 |
| 0NH334Z | 2015 | 2.06 |
| 0NH344Z | 2015 | 2.06 |
| 0NH404Z | 2015 | 2.06 |
| 0NH434Z | 2015 | 2.06 |
| 0NH444Z | 2015 | 2.06 |
| 0NH504Z | 2015 | 2.06 |
| 0NH534Z | 2015 | 2.06 |
| 0NH544Z | 2015 | 2.06 |
| 0NH604Z | 2015 | 2.06 |
| 0NH634Z | 2015 | 2.06 |
| 0NH644Z | 2015 | 2.06 |
| 0NH704Z | 2015 | 2.06 |
| 0NH734Z | 2015 | 2.06 |
| 0NH744Z | 2015 | 2.06 |
| 0NH804Z | 2015 | 2.06 |
| 0NH834Z | 2015 | 2.06 |
| 0NH844Z | 2015 | 2.06 |
| 0NJ00ZZ | 2015 | 1.19 |
| 0NJ03ZZ | 2015 | 1.19 |
| 0NJ04ZZ | 2015 | 1.19 |
| 0NN10ZZ | 2015 | 2.06 |
| 0NN13ZZ | 2015 | 2.06 |
| 0NN14ZZ | 2015 | 2.06 |
| 0NN20ZZ | 2015 | 2.06 |
| 0NN23ZZ | 2015 | 2.06 |
| 0NN24ZZ | 2015 | 2.06 |
| 0NN30ZZ | 2015 | 2.06 |
| 0NN33ZZ | 2015 | 2.06 |
| 0NN34ZZ | 2015 | 2.06 |
| 0NN40ZZ | 2015 | 2.06 |
| 0NN43ZZ | 2015 | 2.06 |
| 0NN44ZZ | 2015 | 2.06 |
| 0NN50ZZ | 2015 | 2.06 |
| 0NN53ZZ | 2015 | 2.06 |
| 0NN54ZZ | 2015 | 2.06 |
| 0NN60ZZ | 2015 | 2.06 |
| 0NN63ZZ | 2015 | 2.06 |
| 0NN64ZZ | 2015 | 2.06 |
| 0NN70ZZ | 2015 | 2.06 |
| 0NN73ZZ | 2015 | 2.06 |
| 0NN74ZZ | 2015 | 2.06 |
| 0NN80ZZ | 2015 | 2.06 |
| 0NN83ZZ | 2015 | 2.06 |
| 0NN84ZZ | 2015 | 2.06 |
| 0NP000Z | 2015 | 1.24 |
| 0NP004Z | 2015 | 1.24 |
| 0NP005Z | 2015 | 1.24 |
| 0NP007Z | 2015 | 1.24 |
| 0NP00JZ | 2015 | 2.07 |
| 0NP00KZ | 2015 | 1.24 |
| 0NP00MZ | 2015 | 1.22 |
| 0NP00NZ | 2015 | 1.29 |
| 0NP00SZ | 2015 | 1.24 |
| 0NP030Z | 2015 | 1.24 |
| 0NP034Z | 2015 | 1.24 |
| 0NP035Z | 2015 | 2.95 |
| 0NP037Z | 2015 | 1.24 |
| 0NP03JZ | 2015 | 2.07 |
| 0NP03KZ | 2015 | 1.24 |
| 0NP03MZ | 2015 | 1.22 |
| 0NP03SZ | 2015 | 1.24 |
| 0NP040Z | 2015 | 1.24 |
| 0NP044Z | 2015 | 1.24 |
| 0NP045Z | 2015 | 2.95 |
| 0NP047Z | 2015 | 1.24 |
| 0NP04JZ | 2015 | 2.07 |
| 0NP04KZ | 2015 | 1.24 |
| 0NP04MZ | 2015 | 1.22 |
| 0NP04SZ | 2015 | 1.24 |
| 0NP0X4Z | 2015 | 1.24 |
| 0NP0X5Z | 2015 | 2.95 |
| 0NP0XMZ | 2015 | 1.22 |
| 0NP0XSZ | 2015 | 1.24 |
| 0NQ00ZZ | 2015 | 2.06 |
| 0NQ03ZZ | 2015 | 2.06 |
| 0NQ04ZZ | 2015 | 2.06 |
| 0NQ0XZZ | 2015 | 2.99 |
| 0NQ10ZZ | 2015 | 2.06 |
| 0NQ13ZZ | 2015 | 2.06 |
| 0NQ14ZZ | 2015 | 2.06 |
| 0NQ1XZZ | 2015 | 2.06 |
| 0NQ20ZZ | 2015 | 2.06 |
| 0NQ23ZZ | 2015 | 2.06 |
| 0NQ24ZZ | 2015 | 2.06 |
| 0NQ2XZZ | 2015 | 2.06 |
| 0NQ30ZZ | 2015 | 2.06 |
| 0NQ33ZZ | 2015 | 2.06 |
| 0NQ34ZZ | 2015 | 2.06 |
| 0NQ3XZZ | 2015 | 2.06 |
| 0NQ40ZZ | 2015 | 2.06 |
| 0NQ43ZZ | 2015 | 2.06 |
| 0NQ44ZZ | 2015 | 2.06 |
| 0NQ4XZZ | 2015 | 2.06 |
| 0NQ50ZZ | 2015 | 2.06 |
| 0NQ53ZZ | 2015 | 2.06 |
| 0NQ54ZZ | 2015 | 2.06 |
| 0NQ5XZZ | 2015 | 2.06 |
| 0NQ60ZZ | 2015 | 2.06 |
| 0NQ63ZZ | 2015 | 2.06 |
| 0NQ64ZZ | 2015 | 2.06 |
| 0NQ6XZZ | 2015 | 2.06 |
| 0NQ70ZZ | 2015 | 2.06 |
| 0NQ73ZZ | 2015 | 2.06 |
| 0NQ74ZZ | 2015 | 2.06 |
| 0NQ7XZZ | 2015 | 2.06 |
| 0NQ80ZZ | 2015 | 2.06 |
| 0NQ83ZZ | 2015 | 2.06 |
| 0NQ84ZZ | 2015 | 2.06 |
| 0NQ8XZZ | 2015 | 2.06 |
| 0NR007Z | 2015 | 2.04 |
| 0NR00JZ | 2015 | 2.05 |
| 0NR00KZ | 2015 | 2.04 |
| 0NR037Z | 2015 | 2.04 |
| 0NR03JZ | 2015 | 2.05 |
| 0NR03KZ | 2015 | 2.04 |
| 0NR047Z | 2015 | 2.04 |
| 0NR04JZ | 2015 | 2.05 |
| 0NR04KZ | 2015 | 2.04 |
| 0NR107Z | 2015 | 2.04 |
| 0NR10JZ | 2015 | 2.05 |
| 0NR10KZ | 2015 | 2.04 |
| 0NR137Z | 2015 | 2.04 |
| 0NR13JZ | 2015 | 2.05 |
| 0NR13KZ | 2015 | 2.04 |
| 0NR147Z | 2015 | 2.04 |
| 0NR14JZ | 2015 | 2.05 |
| 0NR14KZ | 2015 | 2.04 |
| 0NR207Z | 2015 | 2.04 |
| 0NR20JZ | 2015 | 2.05 |
| 0NR20KZ | 2015 | 2.04 |
| 0NR237Z | 2015 | 2.04 |
| 0NR23JZ | 2015 | 2.05 |
| 0NR23KZ | 2015 | 2.04 |
| 0NR247Z | 2015 | 2.04 |
| 0NR24JZ | 2015 | 2.05 |
| 0NR24KZ | 2015 | 2.04 |
| 0NR307Z | 2015 | 2.04 |
| 0NR30JZ | 2015 | 2.05 |
| 0NR30KZ | 2015 | 2.04 |
| 0NR337Z | 2015 | 2.04 |
| 0NR33JZ | 2015 | 2.05 |
| 0NR33KZ | 2015 | 2.04 |
| 0NR347Z | 2015 | 2.04 |
| 0NR34JZ | 2015 | 2.05 |
| 0NR34KZ | 2015 | 2.04 |
| 0NR407Z | 2015 | 2.04 |
| 0NR40JZ | 2015 | 2.05 |
| 0NR40KZ | 2015 | 2.04 |
| 0NR437Z | 2015 | 2.04 |
| 0NR43JZ | 2015 | 2.05 |
| 0NR43KZ | 2015 | 2.04 |
| 0NR447Z | 2015 | 2.04 |
| 0NR44JZ | 2015 | 2.05 |
| 0NR44KZ | 2015 | 2.04 |
| 0NR507Z | 2015 | 2.04 |
| 0NR50JZ | 2015 | 2.05 |
| 0NR50KZ | 2015 | 2.04 |
| 0NR537Z | 2015 | 2.04 |
| 0NR53JZ | 2015 | 2.05 |
| 0NR53KZ | 2015 | 2.04 |
| 0NR547Z | 2015 | 2.04 |
| 0NR54JZ | 2015 | 2.05 |
| 0NR54KZ | 2015 | 2.04 |
| 0NR607Z | 2015 | 2.04 |
| 0NR60JZ | 2015 | 2.05 |
| 0NR60KZ | 2015 | 2.04 |
| 0NR637Z | 2015 | 2.04 |
| 0NR63JZ | 2015 | 2.05 |
| 0NR63KZ | 2015 | 2.04 |
| 0NR647Z | 2015 | 2.04 |
| 0NR64JZ | 2015 | 2.05 |
| 0NR64KZ | 2015 | 2.04 |
| 0NR707Z | 2015 | 2.04 |
| 0NR70JZ | 2015 | 2.05 |
| 0NR70KZ | 2015 | 2.04 |
| 0NR737Z | 2015 | 2.04 |
| 0NR73JZ | 2015 | 2.05 |
| 0NR73KZ | 2015 | 2.04 |
| 0NR747Z | 2015 | 2.04 |
| 0NR74JZ | 2015 | 2.05 |
| 0NR74KZ | 2015 | 2.04 |
| 0NR807Z | 2015 | 2.04 |
| 0NR80JZ | 2015 | 2.05 |
| 0NR80KZ | 2015 | 2.04 |
| 0NR837Z | 2015 | 2.04 |
| 0NR83JZ | 2015 | 2.05 |
| 0NR83KZ | 2015 | 2.04 |
| 0NR847Z | 2015 | 2.04 |
| 0NR84JZ | 2015 | 2.05 |
| 0NR84KZ | 2015 | 2.04 |
| 0NS004Z | 2015 | 2.02 |
| 0NS005Z | 2015 | 2.02 |
| 0NS00ZZ | 2015 | 2.02 |
| 0NS034Z | 2015 | 2.02 |
| 0NS035Z | 2015 | 2.02 |
| 0NS03ZZ | 2015 | 2.02 |
| 0NS044Z | 2015 | 2.02 |
| 0NS045Z | 2015 | 2.02 |
| 0NS04ZZ | 2015 | 2.02 |
| 0NS0XZZ | 2015 | 2.02 |
| 0NS104Z | 2015 | 2.02 |
| 0NS10ZZ | 2015 | 2.02 |
| 0NS134Z | 2015 | 2.02 |
| 0NS13ZZ | 2015 | 2.02 |
| 0NS144Z | 2015 | 2.02 |
| 0NS14ZZ | 2015 | 2.02 |
| 0NS1XZZ | 2015 | 2.02 |
| 0NS204Z | 2015 | 2.02 |
| 0NS20ZZ | 2015 | 2.02 |
| 0NS234Z | 2015 | 2.02 |
| 0NS23ZZ | 2015 | 2.02 |
| 0NS244Z | 2015 | 2.02 |
| 0NS24ZZ | 2015 | 2.02 |
| 0NS2XZZ | 2015 | 2.02 |
| 0NS304Z | 2015 | 2.02 |
| 0NS30ZZ | 2015 | 2.02 |
| 0NS334Z | 2015 | 2.02 |
| 0NS33ZZ | 2015 | 2.02 |
| 0NS344Z | 2015 | 2.02 |
| 0NS34ZZ | 2015 | 2.02 |
| 0NS3XZZ | 2015 | 2.02 |
| 0NS404Z | 2015 | 2.02 |
| 0NS40ZZ | 2015 | 2.02 |
| 0NS434Z | 2015 | 2.02 |
| 0NS43ZZ | 2015 | 2.02 |
| 0NS444Z | 2015 | 2.02 |
| 0NS44ZZ | 2015 | 2.02 |
| 0NS4XZZ | 2015 | 2.02 |
| 0NS504Z | 2015 | 2.02 |
| 0NS50ZZ | 2015 | 2.02 |
| 0NS534Z | 2015 | 2.02 |
| 0NS53ZZ | 2015 | 2.02 |
| 0NS544Z | 2015 | 2.02 |
| 0NS54ZZ | 2015 | 2.02 |
| 0NS5XZZ | 2015 | 2.02 |
| 0NS604Z | 2015 | 2.02 |
| 0NS60ZZ | 2015 | 2.02 |
| 0NS634Z | 2015 | 2.02 |
| 0NS63ZZ | 2015 | 2.02 |
| 0NS644Z | 2015 | 2.02 |
| 0NS64ZZ | 2015 | 2.02 |
| 0NS6XZZ | 2015 | 2.02 |
| 0NS704Z | 2015 | 2.02 |
| 0NS70ZZ | 2015 | 2.02 |
| 0NS734Z | 2015 | 2.02 |
| 0NS73ZZ | 2015 | 2.02 |
| 0NS744Z | 2015 | 2.02 |
| 0NS74ZZ | 2015 | 2.02 |
| 0NS7XZZ | 2015 | 2.02 |
| 0NS804Z | 2015 | 2.02 |
| 0NS80ZZ | 2015 | 2.02 |
| 0NS834Z | 2015 | 2.02 |
| 0NS83ZZ | 2015 | 2.02 |
| 0NS844Z | 2015 | 2.02 |
| 0NS84ZZ | 2015 | 2.02 |
| 0NS8XZZ | 2015 | 2.02 |
| 0NT10ZZ | 2015 | 1.25 |
| 0NT20ZZ | 2015 | 1.25 |
| 0NT30ZZ | 2015 | 1.25 |
| 0NT40ZZ | 2015 | 1.25 |
| 0NT50ZZ | 2015 | 1.25 |
| 0NT60ZZ | 2015 | 1.25 |
| 0NT70ZZ | 2015 | 1.25 |
| 0NT80ZZ | 2015 | 1.25 |
| 0NU007Z | 2015 | 2.04 |
| 0NU00JZ | 2015 | 2.05 |
| 0NU00KZ | 2015 | 2.04 |
| 0NU037Z | 2015 | 2.04 |
| 0NU03JZ | 2015 | 2.05 |
| 0NU03KZ | 2015 | 2.04 |
| 0NU047Z | 2015 | 2.04 |
| 0NU04JZ | 2015 | 2.05 |
| 0NU04KZ | 2015 | 2.04 |
| 0NU107Z | 2015 | 2.04 |
| 0NU10JZ | 2015 | 2.05 |
| 0NU10KZ | 2015 | 2.04 |
| 0NU137Z | 2015 | 2.04 |
| 0NU13JZ | 2015 | 2.05 |
| 0NU13KZ | 2015 | 2.04 |
| 0NU147Z | 2015 | 2.04 |
| 0NU14JZ | 2015 | 2.05 |
| 0NU14KZ | 2015 | 2.04 |
| 0NU207Z | 2015 | 2.04 |
| 0NU20JZ | 2015 | 2.05 |
| 0NU20KZ | 2015 | 2.04 |
| 0NU237Z | 2015 | 2.04 |
| 0NU23JZ | 2015 | 2.05 |
| 0NU23KZ | 2015 | 2.04 |
| 0NU247Z | 2015 | 2.04 |
| 0NU24JZ | 2015 | 2.05 |
| 0NU24KZ | 2015 | 2.04 |
| 0NU307Z | 2015 | 2.04 |
| 0NU30JZ | 2015 | 2.05 |
| 0NU30KZ | 2015 | 2.04 |
| 0NU337Z | 2015 | 2.04 |
| 0NU33JZ | 2015 | 2.05 |
| 0NU33KZ | 2015 | 2.04 |
| 0NU347Z | 2015 | 2.04 |
| 0NU34JZ | 2015 | 2.05 |
| 0NU34KZ | 2015 | 2.04 |
| 0NU407Z | 2015 | 2.04 |
| 0NU40JZ | 2015 | 2.05 |
| 0NU40KZ | 2015 | 2.04 |
| 0NU437Z | 2015 | 2.04 |
| 0NU43JZ | 2015 | 2.05 |
| 0NU43KZ | 2015 | 2.04 |
| 0NU447Z | 2015 | 2.04 |
| 0NU44JZ | 2015 | 2.05 |
| 0NU44KZ | 2015 | 2.04 |
| 0NU507Z | 2015 | 2.04 |
| 0NU50JZ | 2015 | 2.05 |
| 0NU50KZ | 2015 | 2.04 |
| 0NU537Z | 2015 | 2.04 |
| 0NU53JZ | 2015 | 2.05 |
| 0NU53KZ | 2015 | 2.04 |
| 0NU547Z | 2015 | 2.04 |
| 0NU54JZ | 2015 | 2.05 |
| 0NU54KZ | 2015 | 2.04 |
| 0NU607Z | 2015 | 2.04 |
| 0NU60JZ | 2015 | 2.05 |
| 0NU60KZ | 2015 | 2.04 |
| 0NU637Z | 2015 | 2.04 |
| 0NU63JZ | 2015 | 2.05 |
| 0NU63KZ | 2015 | 2.04 |
| 0NU647Z | 2015 | 2.04 |
| 0NU64JZ | 2015 | 2.05 |
| 0NU64KZ | 2015 | 2.04 |
| 0NU707Z | 2015 | 2.04 |
| 0NU70JZ | 2015 | 2.05 |
| 0NU70KZ | 2015 | 2.04 |
| 0NU737Z | 2015 | 2.04 |
| 0NU73JZ | 2015 | 2.05 |
| 0NU73KZ | 2015 | 2.04 |
| 0NU747Z | 2015 | 2.04 |
| 0NU74JZ | 2015 | 2.05 |
| 0NU74KZ | 2015 | 2.04 |
| 0NU807Z | 2015 | 2.04 |
| 0NU80JZ | 2015 | 2.05 |
| 0NU80KZ | 2015 | 2.04 |
| 0NU837Z | 2015 | 2.04 |
| 0NU83JZ | 2015 | 2.05 |
| 0NU83KZ | 2015 | 2.04 |
| 0NU847Z | 2015 | 2.04 |
| 0NU84JZ | 2015 | 2.05 |
| 0NU84KZ | 2015 | 2.04 |
| 0NW000Z | 2015 | 1.24 |
| 0NW004Z | 2015 | 1.24 |
| 0NW005Z | 2015 | 1.24 |
| 0NW007Z | 2015 | 1.24 |
| 0NW00JZ | 2015 | 1.24 |
| 0NW00KZ | 2015 | 1.24 |
| 0NW00MZ | 2015 | 1.24 |
| 0NW00NZ | 2015 | 1.24 |
| 0NW00SZ | 2015 | 1.24 |
| 0NW030Z | 2015 | 1.24 |
| 0NW034Z | 2015 | 1.24 |
| 0NW035Z | 2015 | 1.24 |
| 0NW037Z | 2015 | 1.24 |
| 0NW03JZ | 2015 | 1.24 |
| 0NW03KZ | 2015 | 1.24 |
| 0NW03MZ | 2015 | 1.24 |
| 0NW03SZ | 2015 | 1.24 |
| 0NW040Z | 2015 | 1.24 |
| 0NW044Z | 2015 | 1.24 |
| 0NW045Z | 2015 | 1.24 |
| 0NW047Z | 2015 | 1.24 |
| 0NW04JZ | 2015 | 1.24 |
| 0NW04KZ | 2015 | 1.24 |
| 0NW04MZ | 2015 | 1.24 |
| 0NW04SZ | 2015 | 1.24 |
| 0W110J9 | 2018 | 2.33 |
| 0W110JB | 2018 | 2.33 |
| 0W110JG | 2018 | 2.34 |
| 0W110JJ | 2018 | 2.34 |
| 0W9100Z | 2018 | 1.24 |
| 0W910ZX | 2018 | 1.14 |
| 0W910ZZ | 2018 | 1.24 |
| 0W9130Z | 2018 | 1.09 |
| 0W9130Z | 2018 | 1.24 |
| 0W913ZX | 2018 | 1.13 |
| 0W913ZZ | 2018 | 1.09 |
| 0W913ZZ | 2018 | 1.24 |
| 0W9140Z | 2018 | 1.09 |
| 0W9140Z | 2018 | 1.24 |
| 0W914ZX | 2018 | 1.13 |
| 0W914ZZ | 2018 | 1.09 |
| 0W914ZZ | 2018 | 1.24 |
| 0WC10ZZ | 2018 | 1.24 |
| 0WC13ZZ | 2018 | 1.24 |
| 0WC14ZZ | 2018 | 1.24 |
| 0WF10ZZ | 2018 | 2.92 |
| 0WF13ZZ | 2018 | 2.92 |
| 0WF14ZZ | 2018 | 2.92 |
| 0WH103Z | 2018 | 1.26 |
| 0WH10YZ | 2018 | 1.24 |
| 0WH133Z | 2018 | 1.26 |
| 0WH13YZ | 2018 | 1.24 |
| 0WH143Z | 2018 | 1.26 |
| 0WH14YZ | 2018 | 1.24 |
| 0WJ10ZZ | 2018 | 1.24 |
| 0WJ14ZZ | 2018 | 1.18 |
| 0WP100Z | 2018 | 1.24 |
| 0WP101Z | 2018 | 1.24 |
| 0WP103Z | 2018 | 1.27 |
| 0WP10JZ | 2018 | 1.24 |
| 0WP10YZ | 2018 | 1.24 |
| 0WP130Z | 2018 | 1.24 |
| 0WP131Z | 2018 | 1.24 |
| 0WP133Z | 2018 | 1.27 |
| 0WP13JZ | 2018 | 1.24 |
| 0WP13YZ | 2018 | 1.24 |
| 0WP140Z | 2018 | 1.24 |
| 0WP141Z | 2018 | 1.24 |
| 0WP143Z | 2018 | 1.27 |
| 0WP14JZ | 2018 | 1.24 |
| 0WP14YZ | 2018 | 1.24 |
| 0WP1X3Z | 2018 | 1.27 |
| 0WW100Z | 2018 | 1.24 |
| 0WW101Z | 2018 | 1.24 |
| 0WW103Z | 2018 | 1.24 |
| 0WW10JZ | 2018 | 1.24 |
| 0WW10YZ | 2018 | 1.24 |
| 0WW130Z | 2018 | 1.24 |
| 0WW131Z | 2018 | 1.24 |
| 0WW133Z | 2018 | 1.24 |
| 0WW13JZ | 2018 | 1.24 |
| 0WW13YZ | 2018 | 1.24 |
| 0WW140Z | 2018 | 1.24 |
| 0WW141Z | 2018 | 1.24 |
| 0WW143Z | 2018 | 1.24 |
| 0WW14JZ | 2018 | 1.24 |
| 0WW14YZ | 2018 | 1.24 |
| 3C1ZX8Z | 2018 | 2.41 |
| 3E0Q03Z | 2018 | 1.09 |
| 3E0Q06Z | 2018 | 1.09 |
| 3E0Q07Z | 2018 | 1.09 |
| 3E0Q0AZ | 2018 | 1.09 |
| 3E0Q0BZ | 2018 | 1.09 |
| 3E0Q0E0 | 2018 | 1.09 |
| 3E0Q0E1 | 2018 | 1.09 |
| 3E0Q0GC | 2018 | 1.09 |
| 3E0Q0HZ | 2018 | 1.09 |
| 3E0Q0KZ | 2018 | 1.09 |
| 3E0Q0SF | 2018 | 1.09 |
| 3E0Q0TZ | 2018 | 1.09 |
| 3E0Q33Z | 2018 | 1.09 |
| 3E0Q36Z | 2018 | 1.09 |
| 3E0Q37Z | 2018 | 1.09 |
| 3E0Q3AZ | 2018 | 1.09 |
| 3E0Q3BZ | 2018 | 1.09 |
| 3E0Q3E0 | 2018 | 1.09 |
| 3E0Q3E1 | 2018 | 1.09 |
| 3E0Q3GC | 2018 | 1.09 |
| 3E0Q3HZ | 2018 | 1.09 |
| 3E0Q3KZ | 2018 | 1.09 |
| 3E0Q3SF | 2018 | 1.09 |
| 3E0Q3TZ | 2018 | 1.09 |
| 3E0Q7SF | 2018 | 1.09 |
| 3E1Q38X | 2018 | 2.41 |
| 3E1Q38Z | 2018 | 2.41 |
| 4A003BD | 2018 | 1.1 |
| 4A003KD | 2018 | 1.17 |
| 4A003RD | 2018 | 1.16 |
| 4A007BD | 2018 | 1.1 |
| 4A007KD | 2018 | 1.17 |
| 4A007RD | 2018 | 1.16 |
| 4A008BD | 2018 | 1.1 |
| 4A008KD | 2018 | 1.17 |
| 4A008RD | 2018 | 1.16 |
| 4A103BD | 2018 | 1.1 |
| 4A103KD | 2018 | 1.17 |
| 4A103RD | 2018 | 1.16 |
| 4A107BD | 2018 | 1.1 |
| 4A107KD | 2018 | 1.17 |
| 4A107RD | 2018 | 1.16 |
| 4A108BD | 2018 | 1.1 |
| 4A108KD | 2018 | 1.17 |
| 4A108RD | 2018 | 1.16 |
| 8C01X6J | 2018 | 1.02 |
